# Minimizing the ratio of ionizable lipid in lipid nanoparticles for *in vivo* base editing

**DOI:** 10.1093/nsr/nwae135

**Published:** 2024-04-03

**Authors:** Qiubing Chen, Xuebin Wang, Yizhou Zhang, Ming Tian, Junyi Duan, Ying Zhang, Hao Yin

**Affiliations:** Departments of Urology and Laboratory Medicine, Frontier Science Center for Immunology and Metabolism, Medical Research Institute, Zhongnan Hospital of Wuhan University, Wuhan University, Wuhan 430071, China; State Key Laboratory of Virology, TaiKang Center for Life and Medical Sciences, TaiKang Medical School, Wuhan University, Wuhan 430071, China; Departments of Urology and Laboratory Medicine, Frontier Science Center for Immunology and Metabolism, Medical Research Institute, Zhongnan Hospital of Wuhan University, Wuhan University, Wuhan 430071, China; State Key Laboratory of Virology, TaiKang Center for Life and Medical Sciences, TaiKang Medical School, Wuhan University, Wuhan 430071, China; Departments of Urology and Laboratory Medicine, Frontier Science Center for Immunology and Metabolism, Medical Research Institute, Zhongnan Hospital of Wuhan University, Wuhan University, Wuhan 430071, China; State Key Laboratory of Virology, TaiKang Center for Life and Medical Sciences, TaiKang Medical School, Wuhan University, Wuhan 430071, China; Departments of Urology and Laboratory Medicine, Frontier Science Center for Immunology and Metabolism, Medical Research Institute, Zhongnan Hospital of Wuhan University, Wuhan University, Wuhan 430071, China; State Key Laboratory of Virology, TaiKang Center for Life and Medical Sciences, TaiKang Medical School, Wuhan University, Wuhan 430071, China; Departments of Urology and Laboratory Medicine, Frontier Science Center for Immunology and Metabolism, Medical Research Institute, Zhongnan Hospital of Wuhan University, Wuhan University, Wuhan 430071, China; State Key Laboratory of Virology, TaiKang Center for Life and Medical Sciences, TaiKang Medical School, Wuhan University, Wuhan 430071, China; Departments of Urology and Laboratory Medicine, Frontier Science Center for Immunology and Metabolism, Medical Research Institute, Zhongnan Hospital of Wuhan University, Wuhan University, Wuhan 430071, China; Department of Rheumatology and Immunology, Zhongnan Hospital of Wuhan University, Wuhan University, Wuhan 430071, China; Departments of Urology and Laboratory Medicine, Frontier Science Center for Immunology and Metabolism, Medical Research Institute, Zhongnan Hospital of Wuhan University, Wuhan University, Wuhan 430071, China; State Key Laboratory of Virology, TaiKang Center for Life and Medical Sciences, TaiKang Medical School, Wuhan University, Wuhan 430071, China; Wuhan Research Center for Infectious Diseases and Cancer, Chinese Academy of Medical Sciences, Wuhan 430071, China; RNA Institute, Wuhan University, Wuhan 430071, China

**Keywords:** ionizable lipid, lipid nanoparticle, mRNA delivery, base editor delivery, Pcsk9

## Abstract

Lipid nanoparticles (LNPs) have gained clinical approval as carriers for both siRNA and mRNA. Among the crucial components of LNPs, ionizable lipids play a pivotal role in determining the efficiency of RNA delivery. In this study, we synthesized a series of ionizable lipids, denoted as HTO, with a higher count of hydroxyl groups compared to SM-102. Remarkably, LNPs based on HTO12 lipid demonstrated comparable mRNA delivery efficiency and biosafety to those based on SM-102. However, the former reduced the ratio of ionizable lipid/total lipids to mRNA in LNPs by 2.5 times compared to SM-102. The HTO12 LNP efficiently encapsulated adenine base editor mRNA and sgRNA targeting *Pcsk9*, leading to substantial gene editing within the liver of mice and effective reduction of the target protein. Our study underscores that ionizable lipids with multiple hydroxyl groups may facilitate an improved lipid-to-mRNA ratio to minimize the dosage of ionizable lipids for *in vivo* delivery.

## Teaser

A novel ionizable lipid with multiple hydroxyl groups was synthesized and characterized, demonstrating comparable mRNA delivery efficacy to an ionizable lipid used in clinical settings, while the ratio of ionizable lipid to mRNA in lipid nanoparticles was improved by 2.5 times.

## INTRODUCTION

Gene editing technology has made significant progress for disease treatment [1]. In contrast to Cas9-induced double-strand breaks (DSB) in genomic DNA, base editing tools achieve efficient single base substitutions without introducing DSB [2–7]. Adenine base editor (ABE) consists of an engineered adenine deaminase and Cas9 nickase [2,4]. It can convert adenine (A) in a specific region of the target DNA into inosine (I), which is recognized as guanine (G) on the DNA strand. Consequently, the thymine (T) paired with A on the non-coding strand is converted to cytosine (C), completing the A-to-G or T-to-C replacement. Approximately half of known pathogenic mutations are caused by single nucleotide variations, among which a significant portion requires A to G correction, highlighting the immense potential of base editing tools for gene-based therapy [8].

The broad therapeutic application of base editors relies on efficient *in vivo* delivery, and viral and non-viral vectors have been applied [9,10]. Viral vectors comprise adenovirus (AdV), adeno-associated virus (AAV), lentivirus, and retrovirus [9,10]. Among these, AAV is more widely used due to its low immunogenicity and toxicity [11]. However, the packaging capacity of AAV is relatively small (∼4.7 kb), making the package of all components of base editors in a single virus difficult [9,11–13]. The dual viruses approach has been used for packing base editors; however, it could result in reduced efficiency and increased complexity [14–16]. Furthermore, AAV vectors cannot be applied for repeated injections, further limiting their application [11]. To address these issues, messenger RNA (mRNA)-based non-viral vectors have been used for preclinical and clinical development to deliver base editors [17–20].

The development of mRNA-based therapeutics has emerged as a groundbreaking approach for treating and preventing various diseases, including genetic disorders, infectious diseases, and cancer [21–23]. The use of mRNA holds significant promise due to its ability to produce therapeutic proteins inside cells, enabling precise and customizable interventions at the molecular level. However, the translation of mRNA-based therapies from bench to bedside has faced substantial challenges, predominantly related to efficient delivery of exogenous mRNA molecules into target cells. Effective mRNA delivery demands the circumvention of numerous biological barriers, including enzymatic degradation, poor cellular uptake, and innate immune responses [24]. To address these hurdles, significant efforts have been devoted to the development of delivery systems, with lipid nanoparticles (LNPs) emerging as a particularly promising category [25–30]. As of now, several LNP-based nucleic acid drugs have been approved by the FDA, including ONPATTRO® (patisiran), a siRNA therapy to treat polyneuropathy caused by hereditary transthyretin-mediated amyloidosis (hATTR) [31], as well as two mRNA-based COVID-19 vaccines, mRNA-1273 and BNT162b21 [32,33]. The original LNPs are composed of four components: an ionizable lipid, a phospholipid, cholesterol, and lipid-conjugated polyethylene glycol (PEG) [29,30]. The ionizable lipid is considered the most critical component, which exhibits pH-responsive characteristics, remaining uncharged in neutral environments and acquiring a positive charge in acidic environments, allowing them to effectively encapsulate RNA by binding to negatively charged RNA [29,30]. The variable charge feature of ionizable lipids ensures that LNPs are uncharged in neutral environments, thereby reducing their toxicity during circulation in the bloodstream [29,30]. Moreover, they acquire a positive charge in the acidic microenvironment of endosomes, significantly enhancing LNP's ability to escape lysosomes and promoting the release of RNA into the cytoplasm [29,30].

Despite the fact that ionizable lipids are considered to have lower immunogenicity than cationic lipids, clinical trials have shown that subjects still experience varying degrees of adverse reactions [31,34,35]. For instance, ONPATTRO® holds adverse side effects such as rash, discomfort, nausea, abdominal pain, expiratory dyspnea, and headache [31]. To mitigate these effects, subjects received corticosteroids, acetaminophen, and antihistamines as pre-treatment [31]. Two mRNA-based COVID-19 vaccines, mRNA-1273 and BNT162b21, administered through intramuscular injection, have led to side effects like pain, swelling, fever, and sleepiness [34,35]. Some subjects also experienced delayed hypersensitivity reactions, myocarditis/pericarditis, and central nervous system inflammation [34,35]. Although specific triggers of these side effects remain unclear, ionizable lipids possess inflammatory properties [36]. In rodents, elevated levels of various inflammatory factors (IL-1b, GM-CSF, IL6, etc.) and chemokines (CCL2, CCL3, CXCL1, CXCL2, etc.) were observed, along with significant neutrophil infiltration at the injection sites via either subcutaneous or intramuscular routes, and the removal of ionizable lipids from the LNPs eliminated such phenomena [36]. Hence, a promising approach to ameliorate the undesirable effects triggered by ionizable lipids in mRNA-LNP formulations is to decrease the dosage of these lipids or to develop safer alternatives.

In this study, we designed a new set of ionizable lipids, through increasing the number of hydrogen bond donors in these compounds. This modification led to improved payload capacity and reduced the amount of lipid composition in LNP, in comparison to SM-102, a clinically validated ionizable lipid that has been applied as the key component in COVID-19 vaccines [29,32,37–39]. SM-102 efficiently targets the liver after intravenous injection of its formulated LNP [29,32,37–39]. The HTO LNPs exhibited excellent stability, safety, and mRNA delivery efficiency, comparable to SM-102, while the HTO LNP contained 2.5-fold less ionizable lipid/total lipids than SM-102. Subsequently, HTO LNP achieved effective co-delivery of ABE mRNA and sgRNA, reaching an editing efficiency of 63.4% after a single injection and effectively reducing the expression of proprotein convertase subtilisin/kexin type 9 (Pcsk9) protein. Our study indicates optimal mRNA delivery can be achieved by using a lower than usual amount of ionizable lipid, and highlights a path to minimize the ratio of the ionizable lipid in LNPs.

## RESULTS

### Design and synthesis of new ionizable lipids

**Figure 1.**
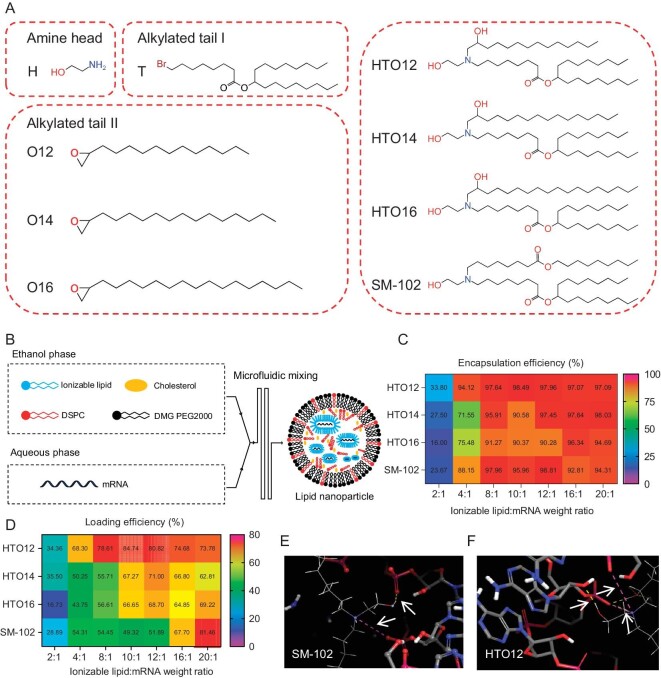
Rational design and screening of HTO LNP. (A) Chemical structures of ionizable lipid HTO12, HTO14 and HTO16, and the corresponding starting reactants. (B) Schematic illustration of LNPs preparation using a microfluidics device. (C and D) The mRNA encapsulation efficiency (C) and drug loading efficiency (D) with various weight ratios of ionizable lipid and mRNA. (E and F) Molecular dynamics simulation of hydrogen bonding forces between RNA and SM-102 (E) and HTO12 (F).

We designed and synthesized a class of multi-hydroxyl ionizable lipids [32]. The hydroxyl groups were situated at the head of ethanolamine and one of the alkyl chains, respectively (Fig. 1A and Fig. S1). The hydroxyl groups on the alkyl chain were obtained through epoxide ring-opening reactions, resulting in three new ionizable lipids with side chains of different lengths (named HTO12, HTO14, and HTO16). Further details regarding the synthesis processes can be found in the Supplementary information. The lipid structures were confirmed using ^1^H nuclear magnetic resonance (NMR) and liquid chromatography coupled to mass spectrometry (LC-MS) (Figs S2–S12).

### Characterization of the ionizable LNPs

Ionizable lipid-based LNPs were formulated using a microfluidic mixing device, following a previously established protocol [38]. The formulation comprised ionizable lipid, helper lipid (DSPC), cholesterol, and a PEG-lipid conjugate (DMG-PEG2000) with a molar ratio of 50:10:38.5:1.5 (Fig. 1B) [38]. We used Firefly luciferase (Fluc) mRNA to measure the efficiency of *in vitro* and *in vivo* delivery [38,40]. We examined various weight ratios of ionizable lipid to mRNA ranging from 2:1 to 20:1. The encapsulation efficiency and drug (mRNA) loading capacity are used as important criteria for evaluating the quality of LNPs. Encapsulation efficiency signifies the ratio of RNA enclosed within lipid nanoparticles to the total RNA within the LNP solution, whereas a high encapsulation efficiency indicates a low presence of free mRNA in the prepared LNP solution. On the other hand, drug loading efficiency measures the proportion of RNA encapsulated in lipid nanoparticles concerning the RNA input during the preparation process. Consequently, a high drug loading efficiency reflects a relatively minor loss of mRNA in LNP throughout the preparation process. At ionizable lipid to mRNA weight ratios exceeding 2:1, the encapsulation efficiency of LNPs generated from three HTO lipids and SM-102 all surpassed 70% (Fig. 1C). Notably, the drug (mRNA) loading efficiency of HTO LNP is superior to that of SM-102 (Fig. 1D). Furthermore, it was observed that LNPs exhibited negative surface charges at neutral pH, potentially due to the presence of negatively charged mRNA molecules (Table S1) [18,41]. The molecular dynamics simulations suggested that the presence of more hydroxyl groups in HTO lipids could form more hydrogen bonds with mRNA than SM-102 (Fig. 1E and F). These interactions may enhance the binding affinity between lipid and mRNA, thereby reducing the loss of mRNA during LNP preparation.

### *In vivo* screening of formulations

**Figure 2.**
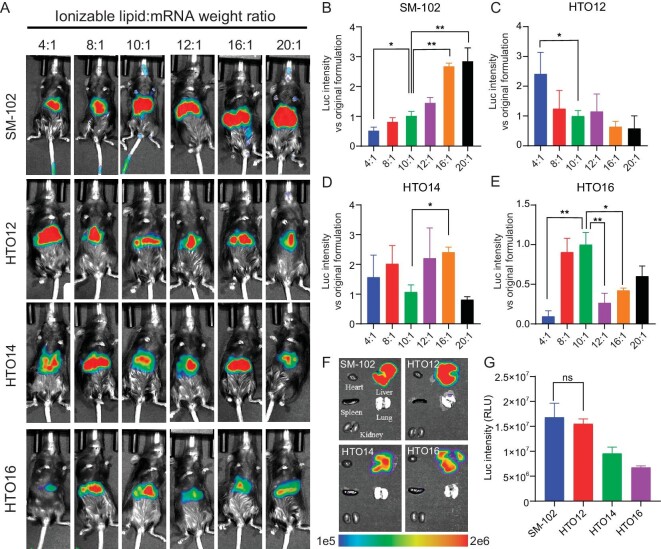
(A) *In vivo* whole-body luminescence imaging after delivery of SM-102, HTO12, HTO14 and HTO16 LNPs encapsulating luciferase-encoding mRNA at a dose of 0.5 mg/kg. (B–E) Normalized average luminescence of SM-102 (B), HTO12 (C), HTO14 (D) and HTO16 (E) LNPs against the standard formulation group (ionizable lipid:mRNA as 10:1, weight/weight) after 6 h of injection. (F) Bioluminescent imaging of representative tissues. (G) Quantified average luminescence of the liver tissues. Each point represents the mean ± SD (*n* = 3). *P < 0.05, **P < 0.01, ns, no significance.

Previous findings have indicated that hydroxyl groups on ionizable lipids could significantly improve mRNA delivery efficiency *in vivo*, while the ratio of ionizable lipid to mRNA remains as standard of lipid to mRNA ratio of 10:1 (weight/weight) [39,42]. We hypothesized that reducing the ratio of ionizable lipids in LNP may be possible for lipids with sufficient hydroxyl groups. *In vivo* screening of LNPs for mRNA delivery was conducted via chemiluminescence imaging of rodents, and we also conducted a screening of the SM-102 formulation (Fig. 2A and B). The lipid to mRNA ratio for the standard formulation of SM-102 is 10:1 (weight/weight) [39]. Reducing the lipid ratio of SM-102 from 10:1 significantly reduced the delivery efficiency of mRNA (Fig. 2A and B). In contrast, for HTO12 lipid, the 4:1 (weight/weight) ratio showed the highest expression efficiency, being 2.5 times improved than that of 10:1 (Fig. 2A and C). In addition, the other two HTO LNPs (HTO14 and HTO16) did not display a ratio-dependent increase of delivery efficiency as that of SM-102 (Fig. 2A, D and E). The selected optimal formulations have been used for subsequent experiments (Table S1). For analysis of LNP biodistribution, we collected mouse tissues for imaging. HTO LNPs effectively delivered mRNA to the liver, which aligns with SM-102 LNP (Fig. 2F). Other organs showed negligible translation of Fluc protein. Moreover, the levels of luciferase expression were similar between the HTO12 group and the SM-102 group, while HTO12 LNP contained 2.5-fold less ionizable lipid than the SM-102 group (Fig. 2G).

We conducted a comprehensive characterization of the physicochemical properties utilizing the optimal HTO12, HTO14, HTO16, and SM-102 LNPs, including particle size, polydispersity index (PDI), zeta potential, pKa, and stability (Figs S13 and S14, Table S1). All LNPs exhibited a consistent average particle size within the range of 60 to 65 nm, a low PDI of less than 0.15 and a negative zeta potential (−9 to −10 mV) in PBS solution (Table S1). The pKa values of HTO LNPs were observed to range from 6.4 to 6.5 (Fig. S13). Both HTO12 LNP and SM-102 LNP exhibited substantial stability to maintain their integrity for a duration of four weeks in PBS at 4°C (Fig. S14A and B) and 6 h in 10% FBS at 37°C (Fig. S14C and D) [43]. Concurrently, it was observed that under identical storage conditions, both HTO12 LNP and SM-102 LNP exhibited sustained and comparable ability of *in vivo* mRNA delivery (Fig. S14E and F). This outcome underscores that reducing HTO lipids input does not compromise the stability of the LNP in aqueous solution.

### Biosafety of the optimized LNPs

**Figure 3.**
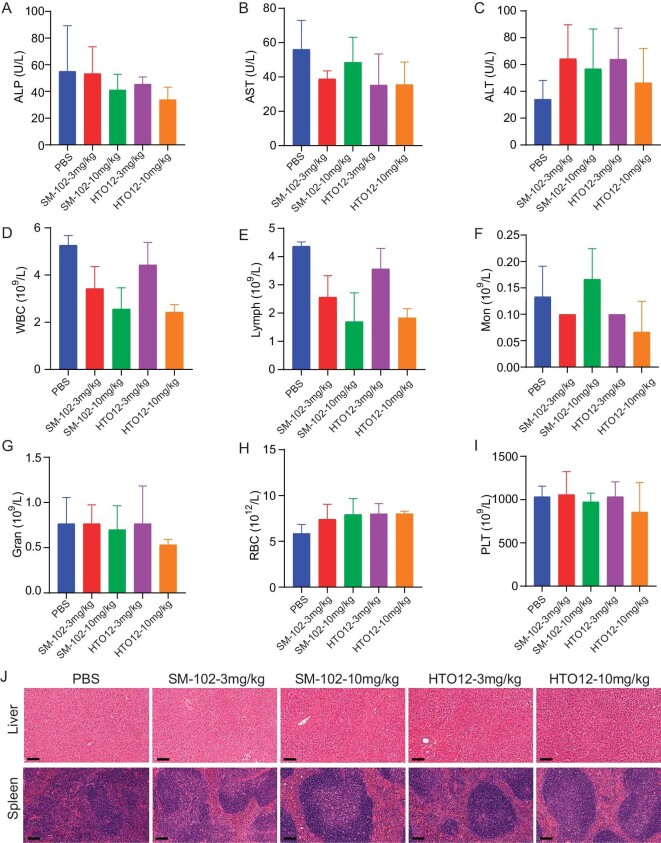
Biosafety evaluation of blank SM-102 and HTO12 LNP. (A–C) The concentrations of ALP (A), AST (B), and ALT (C) in plasma from mice treated with different concentrations of empty SM-102 and HTO12 LNP. (D–I) Evaluation of the standard hematology markers, including white blood cell (WBC) (D), lymphocyte (Lymph) (E), monocytes (Mon) (F), neutrophilic granulocyte (Gran) (G), red blood cell (RBC) (H) and platelet (PLT) (I). (J) H&E staining of the liver and spleen. Scale bar: 100 μm. Each point represents the mean ± SD (*n* = 3).

We conducted a comprehensive biosafety analysis of HTO12 LNP. SM-102, the lipid that has been well-documented and widely used in humans, served as a control. The dosage standard for blank LNP was based on the theoretically equivalent mRNA levels. Even when mice were administered high doses of LNP (equivalent to 10 mg/kg mRNA), there was no significant increase in transaminase levels in mouse plasma related to liver toxicity (Fig. 3A–C). The blood test revealed that various indicators were within the normal range for both HTO12 and SM-102 groups (Fig. 3D–I). Additionally, we examined the histology of the major organs that LNPs target, and no significant lesions were found (Fig. 3J). We have injected total lipids doses up to 200 mg/kg, which has reached the upper limit of lipid dose used. It is difficult to inject a total lipids dose more than 200 mg/kg for mice. A biosafety study in non-human primates would provide more relevant information. Injecting equivalent doses of HTO12 LNP and SM-102 LNP intramuscularly (2 μg mRNA per injection site) did not reveal any aberrant expression of examined chemokines (*Ccl2, Ccl7* and *Cxcl10*) or cytokines (*Gm-csf, Il6* and *Tnf-α*) within the tissue surrounding the injection site, suggesting neither HTO12 LNP nor SM-102 LNP induces substantial inflammation response under physiologically relevant doses (Fig. S15). These data collectively suggested that HTO12 LNP demonstrated a biosafety profile comparable to that of SM-102.

### Evaluation of base editing efficiency *in vivo*

**Figure 4.**
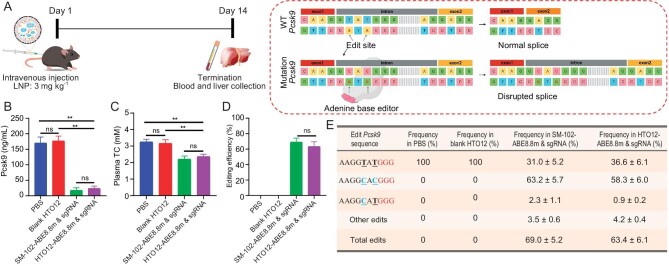
(A) Schematic of ABE mediated *Pcsk9* exon 1 mutation to produce disrupted exon splicing (created with BioRender.com). (B–E) The levels of serum Pcsk9 protein (B) and total cholesterol (C) in mice injected with PBS, blank HTO12 LNP, SM-102 or HTO12 LNPs encapsulating ABE (ABE8.8 m encoding mRNA and an sgRNA targeting *Pcsk9*). (D and E) Editing efficiencies and outcomes in the liver induced by LNP treatment. Data were generated by amplicon sequencing. Each point represents the mean ± SD (*n* = 5). *P < 0.05, **P < 0.01, ns, no significance.

Disordered cholesterol metabolism has been implicated in the pathogenesis of various illnesses, including atherosclerosis and familial hypercholesterolemia [44,45]. Over recent years, PCSK9 has emerged as a key target of drug development for mitigating dyslipidemia [44,45]. PCSK9 facilitates low-density lipoprotein receptor (LDLR) degradation, thereby blocking the function or expression of PCSK9 reduces plasma LDL cholesterol levels [44,45]. ABE has been used to reduce the expression of target proteins by disrupting pre-mRNA splicing sites [17,20]. We used an sgRNA (named as es2 sgRNA) targeting the first splicing site (between exon 1 and intron) in the *Pcsk9* gene [17,20]. The protospacer adjacent motif (PAM) sequence is located on the intron sequence (Fig. 4A). To improve the translation of ABE mRNA *in vivo*, we optimized the sequence of ABE by synonymous codon substitution to reduce the content of uridine (U) in the sequence, introduced N1-methyl-pseudouridine modification, and removed double-stranded RNA (dsRNA) generated during *in vitro* transcription (IVT) [28].

We formulated HTO12 LNPs to co-deliver ABE mRNA and es2 sgRNA following the methods described above. The size of HTO12 LNPs carrying ABE mRNA and es2 sgRNA remained at 67 nm, consistent with HTO12 LNP encapsulating Fluc mRNA (Fig. S16A). Cryogenic-transmission electron microscopy (Cryo-TEM) micrographs confirmed the spherical shape of LNPs (Fig. S16B). Subsequently, we evaluated the *in vivo* delivery capability of HTO12 LNPs for ABE. We treated rodents with blank HTO12 LNP (LNP without encapsulating ABE), SM-102 or HTO12 LNPs encapsulating ABE (mRNA encoding ABE8.8m and a *Pcsk9* targeting sgRNA). HTO12 LNP encapsulating ABE exhibited an average reduction of 86.6% in Pcsk9 protein and a reduction level of 25.2% in total cholesterol, which was comparable to efficacy by SM-102 LNP (Fig. 4B and C). Concurrently, we observed that blank HTO12 LNP exhibited no discernible influence on plasma Pcsk9 and cholesterol levels when compared against the control group administered with PBS (Fig. 4B and C). Subsequently, deep sequencing analysis of the genomic DNA from the livers of HTO12 LNP encapsulating ABE treated animals showed an average of 63.4% editing efficiency, also comparable to that of the SM-102 LNP group (Fig. 4D and E).

## DISCUSSION

Base editors hold great potential in treating genetic diseases [3,4]. Viral vectors have been examined in rodents for delivery of base editors, while these vectors have restricted cargo capacity for base editors, and they often induce immune responses. Non-viral delivery of base editor has been proven effective in mice and non-human primates, through co-delivery of mRNA encoding a base editor and one sgRNA targeting the disease-related gene, both of which are encapsulated in LNP [17,19,20]. Despite the initial success in rodents and non-human primates, intravenous administration of a higher dose of LNP than delivery of mRNA encoding a therapeutic protein or even Cas9 for indel formation, is usually required for delivery of base editors [17,19,20,28,46]. This requirement of high dose for precision editors limits the broad usage of base editors or even prime editors for gene-based therapies. Therefore, more effective LNPs are required for delivery of precision editors [21,26,27,29,30].

The formation of lipid nanoparticles primarily entails the protonation of ionizable lipids within acidic environments, subsequently facilitating their binding to negatively charged mRNA molecules [21,26,27,29,30]. Furthermore, the presence of weak interactions between ionizable lipids and RNA, notably hydrogen bonds, has been identified [42,47]. It was shown that hydrogen bonding interactions between the hydroxyl headgroup of lipids and mRNA play a crucial role in contributing to the *in vivo* expression of mRNA-LNPs [42]. Notably, two distinct ionizable lipids, SM-102 and ALC-0315, employed in the preparation of COVID-19 vaccines, exhibit a structurally similar characteristic, which both have the hydroxyl headgroup [29]. Additionally, lipid 29, a lipid molecule designed and synthesized by Moderna, features an increased number of hydrogen bonding donors/receptors within the square amide moiety of its headgroup, thereby demonstrating superior *in vivo* mRNA delivery efficiency compared to lipids with only a single hydroxyl group [47]. These findings suggest that an increasing hydrogen bonding force between ionizable lipids and mRNA could enhance the delivery efficiency of mRNA-LNPs. However, it has not yet been demonstrated whether increasing hydrogen bonding could lead to a modification in the formulation of LNP [25,29,39,42,47].

In this study, we successfully synthesized a class of ionizable lipids through the ring-opening reaction of an epoxyalkane with ethanolamine. These lipids, named as HTO, contain multiple hydroxyl groups. Compared to the FDA-approved SM-102, HTO12 exhibited a similar efficiency and biosafety profile in delivering mRNA while using a much lower amount of lipid in LNP formulation with improved drug (mRNA) loading capacity of LNPs. HTO12 effectively co-encapsulated mRNA encoding a large adenine base editor (more than 5000 bp) and an sgRNA (100 bp) targeting *Pcsk9*, achieving a reduction of Pcsk9 protein and serum total cholesterol. Collectively, with these remarkable efficacy and safety profiles, as well as reduced usage of lipid and improved drug (mRNA) loading efficiency, we believe HTO12 holds significant promise for mRNA therapeutics. Applying the knowledge in this study may guide the design of new ionizable lipids with reduced lipid ratios in LNP, favoring the development of next-generation carriers for gene-based therapies.

## ETHICAL STATEMENT

The animal experiments in this study were carried out according to the laboratory animal welfare Chinese National Standard (GB/T 35892-2018).

## Supplementary Material

nwae135_Supplemental_File

## Data Availability

The data that support the findings of current study are available within this manuscript and under National Center for Biotechnology Information BioProject accession no. PRJNA1010958.

## FUNGDING

This work was supported by the National Key R&D Program of China (2019YFA0802801, 2018YFA0801401 and 2022YFF1002801), the Key R&D Program of Hubei Province (2022BCA089 to H.Y. and 2022ACA005 to Y.Z.), the Ministry of Agriculture and Rural Affairs of China, the National Natural Science Foundation of China (31871345 and 32071442 to H.Y., 31972936 to Y.Z.), the State Key Laboratory for Animal Disease Control and Prevention (SKLVBF202202), the Medical Science Advancement Program (Basic Medical Sciences) of Wuhan University (TFJC2018004), the Fundamental Research Funds for the Central Universities (2042022dx0003 and 2042022kf1190), the Applied Basic Frontier Program of Wuhan City (2020020601012216 to H.Y.) and the startup funding from Wuhan University (to H.Y. and Y.Z.).
