# Supplementary material for: Minimizing the ratio of ionizable lipid in lipid nanoparticles for in vivo base editing
Source: Natl Sci Rev. 2024 Apr 3;11(6):nwae135. doi: 10.1093/nsr/nwae135 (PMC11104531; doi:10.1093/nsr/nwae135)
Supplement: nwae135_Supplemental_File [file nwae135_supplemental_file.pdf]

Supplemental Information

Supplemental Materials and Methods

Materials

DSPC and Cholesterol were purchased from A.V.T. Pharmaceutical Co. Ltd (Shanghai, China). SM-102 and DMG-PEG2000 was purchased from Xiamen Sinopeg Biotech Co., Ltd. (Xiamen, China). Cap-2'-O-methyltransferase and Vaccinia capping enzyme was purchased from New England Biolabs (MA, USA). Diagnostic kits to determine Alkaline phosphatase (ALP), glutamic-pyruvic transaminase (ALT), and glutamic-oxaloacetic transaminase (AST) were purchased from Beijing Boxbio Science & Technology Co., Ltd (Beijing, China). rNTP mix was purchased from Novoprotein Scientific Inc. (Shanghai, China). Acetate buffer solution was purchased from Coolaber Science & Technology (Beijing, China). Quant-iT™ RiboGreen™ kit was purchased from Thermo Fisher Scientific Inc. (Cleveland, OH, USA). Mouse Pcsk9 ELISA kit was purchased from Proteintech Group Inc. (Wuhan, China). D-Luciferin potassium was purchased from Solarbio Life Sciences (Beijing, China). Total cholesterol (TC) Kit was purchased from Shanghai Kehua Bio-Engineering Co., Ltd. (Shanghai, China). PBS and Triton X-100 were purchased from Adamas-beta® (Shanghai, China). T7 High Yield RNA Transcription kit, HiScript II 1st strand cDNA synthesis kit and ChamQ SYBR qPCR Master Mix were purchased from Vazyme (Nanjing, China). 1,2-epoxytetradecane (denoted as O12-1), 1,2-epoxyhexadecane (denoted as O14-1), 1,2-epoxyoctadecane (denoted as O16-1), 8-bromooctanoic acid (denoted as compound 1), 9-heptadecanol (denoted as compound a) and ethanolamine were purchased from Macklin Biochemical Technology Co., Ltd. (Shanghai, China). 2-(p-toluidinyl) naphthalene-6-sulfonic acid (TNS) probe was purchased from Sigma-Aldrich (MO, USA).

Synthesis of Ionizable Lipid

Synthesis and characterization of shared lipid 3

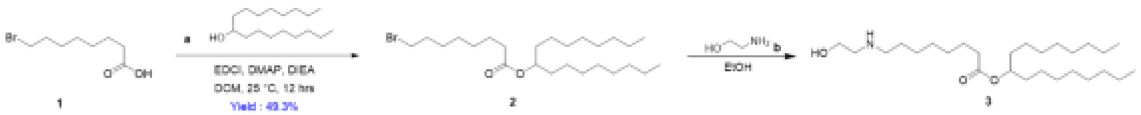

To a solution of compound 1 (100 g, 448 mmol, 1.00 eq) in dichloromethane (DCM) (1500 mL) was added compound a (126.5 g, 493 mmol, 1.10 eq) and 1-Ethyl-3-(3-dimethylaminopropyl) carbodiimide (EDCI) (103 g, 537 mmol, 1.20 eq) and 4-Dimethylaminopyridine (DMAP) (11.0 g, 89.6 mmol, 0.20 eq) and N, N-Diisopropylethylamine (DIEA) (231 g, 1.79 mol, 312 mL, 4.00 eq). The mixture was stirred at 25 ℃ for 12 h. Thin-layer chromatography (TLC) (Plate 1 : Petroleum ether:Ethyl acetate = 5:1, Color Developing Reagent : Iodine colorimetry) indicated Reactant 1 (Rf = 0.20) was consumed completely and many new spots (Rf = 0.56, 0.90) formed. The LCMS nonsense. The reaction mixture was concentrated under reduced pressure to remove DCM. The residue was diluted with Ethyl acetate (EtOAc) 1000 mL and organic layer was washed with brine 1000 mL (500 mL × 2), dried over [Na_2SO_4], filtered and concentrated under reduced pressure to give a residue. The residue was purified by flash silica gel chromatography (ISCO®; 250 g SepaFlash® Silica Flash Column, Eluent of 0 ~ 1% Ethyl acetate / Petroleum ether). TLC: Plate 2: Petroleu mether: Ethyl acetate = 20:1, Color Developing Reagent: Iodine colorimetry, desired Rf = 0.62. Compound 2 (102 g, 221 mmol, 49.3% yield) was obtained as a lightly yellow oil which was confirmed by HNMR (Figure S2).

A solution of Compound 2 (2.00 g, 4.33 mmol, 1.00 eq) and a (5.29 g, 86.6 mmol, 5.24 mL, 20.0 eq) in Methanol (MeOH) (56 mL) was heated to 50 ℃ and stirred at 16 h. LCMS showed the Compound 2 was consumed and the desired mass (RT = 0.672 mins) was detected. TLC (petroleum ether/ ethyl acetate = 5/1, I_2) showed the Compound 2 (Rf = 0.50) was consumed and one new spot was formed. TLC (DCM/MeOH = 5/1, I_2) showed the Compound 2 (Rf = 1.00) was consumed and one new spot (Rf = 0.45) was formed. The residue was dissolved in DCM (20.0 mL) washed by sodium bicarbonate solution (10.0 mL), washed by brine (10.0 mL), dried

over Na_2SO_4, the organic phase was concentrated under reduced pressure to give a residue. The residue was purified by flash silica gel chromatography (ISCO®; 5.00g SepaFlash® Silica Flash Column, eluent of 0~50% DCM/MeOH, Rf = 0.45) to get Compound 3 (1.16 g, 2.62 mmol, 60.5% yield) as yellow oil, which was confirmed by HNMR (Figure S3).

Synthesis and characterization of lipid HTO12

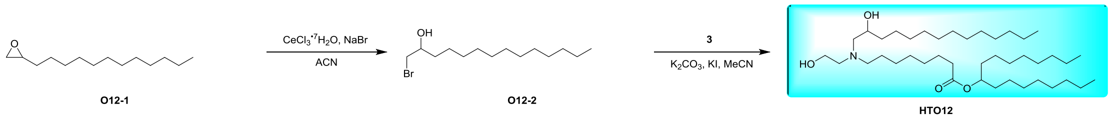

To a solution of O12-1 (800 mg, 3.77 mmol, 1 eq) in Acetonitrile (ACN) (47.0 mL) was added NaBr (457 mg, 4.45 mmol, 142 μL, 1.18 eq) followed by trichlorocerium (1.10 g, 4.45 mmol, 279.5 μL, 1.18 eq), then the reaction was stirred at 20 ℃ for 18 h. TLC (PE/EA=5/1, I_2) showed the O12-1 (Rf = 0.50) was consumed and the one new spot (Rf = 0.30) was formed. The reaction mixture was concentrated to remove ACN, then added H_2O (50.0 mL) and extracted three times with ethyl acetate (150 mL total). The EA fractions we combined and concentrated under reduced pressure to get residue. The residue was purified by flash silica gel chromatography (ISCO®; 1.00g SepaFlash® Silica Flash Column, eluent of 0~50% DCM/MeOH, Rf = 0.30) to get O12-2 (788 mg, 2.69 mmol, 71.33% yield) as colorless oil, which was confirmed by HNMR (Figure S4). HNMR: δ 3.77 (qd, J = 6.48, 3.31 Hz, 1H), 3.54 (dd, J = 10.26, 3.25 Hz, 1H). 3.38 (dd, J = 10.32, 7.07 Hz, 1H), 2.21 (br d, J = 5.88 Hz, 1H), 1.50 - 1.61 (m, 2H), 1.24 - 1.34 (m, 19H), 0.85 - 0.91 (m, 3H).

To a mixture of O12-2 (117 mg, 398 μmol, 1.00 eq) and Compound 3 (176.2 mg, 398 μmol, 1.00 eq) in ACN (3.75 mL) under dry nitrogen was added KI (72.8 mg, 438 μmol, 1.10 eq) followed by K_2CO_3 (220 mg, 1.60 mmol, 4.00 eq) and the mixture diluted with methoxycyclopentane (344 mg, 3.44 mmol, 401 μL, 8.63 eq). The resulting white mixture was heated to 90 ℃ and stirred for 28 h, TLC (DCM/MeOH=10/1, PMA) showed Compound 3 (Rf = 0.50) was consumed and one

new main spot (Rf = 0.60) was formed. The residue was filtered, the filter was washed by DCM (15.0 mL), the precipitate was filtered off and the filtrate was washed by $NaHCO_3$ (5mL × 2), dried over Na_2SO_4, the organic phase was concentrated under reduced pressure to get residue. The residue was purified by flash silica gel chromatography (ISCO®; 1.00g SepaFlash® Silica Flash Column, eluent of 0~50% DCM/MeOH, Rf = 0.60) to get HTO12 (200 mg, 299 μmol, 7.51% yield, 98% purity) as yellow oil, which was confirmed by HNMR (Figure S5) and LCMS (Figure S6). HNMR: δ 4.87 (J = 6.19 Hz, 1H), 3.59 - 3.76 (m, 3H), 2.76 - 2.85 (m, 1H), 2.58 - 2.67 (m, 2H), 2.49 - 2.57 (m, 2H), 2.40 - 2.46 (m, 1H), 2.28 (br t, J = 7.50 Hz, 2H), 1.61 (br d, J = 6.88 Hz, 2H), 1.45 - 1.55 (m, 8H), 1.21 - 1.36 (m, 50H), 0.89 (t, J = 6.75 Hz, 9H).

Synthesis and characterization of lipid HTO14

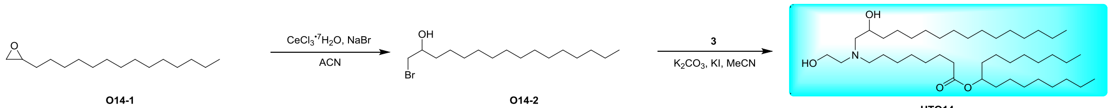

To a solution of O14-1 (1.5 g, 6.24 mmol, 1 eq) in ACN (100 mL) was added NaBr (757 mg, 7.36 mmol, 236 μL, 1.18 eq) followed by trichlorocerium (1.81g, 7.36 mmol, 462 μL, 1.18 eq), then the reaction was stirred at 20 ℃ for 18 h. TLC (PE/EA=5/1, I_2) showed the O14-1 (Rf = 0.60) was consumed and the two new spot (Rf = 0.70 and 0.45) were formed. The reaction mixture was concentrated to remove ACN, then added H_2O (100.0 mL) and extracted three times with ethyl acetate (200 mL total). The EA fractions we combined and concentrated under reduced pressure to get residue. The residue was purified by flash silica gel chromatography (ISCO®; 2.00g SepaFlash® Silica Flash Column, eluent of 0~50% PE/EA, Rf = 0.45) to get O14-2 (1.4 g, 4.36 mmol, 69.83% yield) as white solid, which was confirmed by HNMR (Figure S7).

To a mixture of O14-2 (500 mg, 1.56 mmol, 1.00 eq) and Compound 3 (687 mg, 1.56 mmol, 1.00 eq) in ACN (10 mL) under dry nitrogen was added KI (258 mg, 1.56 mmol, 1.00 eq) followed by K_2CO_3 (860 mg, 6.22 mmol, 4.00 eq). The resulting white mixture was heated to 90 ℃ and stirred

for 20 h. LCMS displays~76.5% of O14-2 (Rt = 0.574 min) remaining, ~22.3% of Compound 3 mass value of 25 (Rt = 0.714 min) was detected. Concentrate the reaction solution directly to remove acetonitrile, dilute with 100 mL of ethyl acetate, and wash twice with saturated salt water (100 mL × 2). The organic phase is dried with anhydrous sodium sulfate and filtered and concentrated to obtain the crude product. The crude product is purified by gradient elution on a silica gel column (ISCO®; 2.00 g SepaFlash® Silica Flash Column, Ellent of 0~3% Dichloromethane/Methanol, TLC: Dichloromethane/Methanol=10/1, Rf=0.58) yields a yellow oily target product HTO14 (0.26 g, 375 μmol, 24.1% yield, 98.4% purity), validated by HNMR (Figure S8) and LCMS (Figure S9). HNMR: δ = 4.86 (quin, J = 6.2 Hz, 1H), 3.58-3.73 (m, 3H), 2.78 (ddd, J = 4.8, 8.0, 13.2 Hz, 2H), 2.56-2.68 (m, 2H), 2.46-2.55 (m, 2H), 2.35-2.46 (m, 1H), 2.28 (t, J = 7.6 Hz, 2H), 1.57-1.68 (m, 2H), 1.40-1.54 (m, 8H), 1.18-1.37 (m, 55H), 0.88 (t, J = 6.8 Hz, 9H).

Synthesis and characterization of lipid HTO16

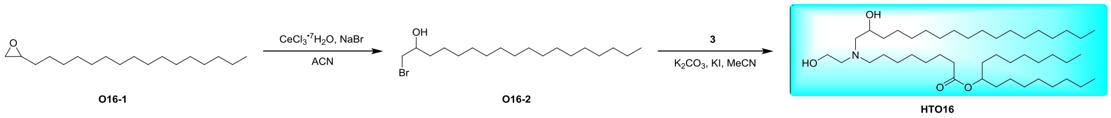

To a solution of O16-1 (2.00 g, 7.45 mmol, 1.00 eq) in ACN (118 mL) was added NaBr (904 mg, 8.79 mmol, 282 μL, 1.18 eq) followed by trichlorocerium (2.17 g, 8.79 mmol, 552 μL, 1.18 eq), then the reaction was stirred at 20 ℃ for 12 h. TLC (PE/EA = 5/1, I_2) showed the O16-1 (Rf = 0.50) was consumed and the one new spot (Rf = 0.35) was formed. The reaction mixture was concentrated, then added H_2O (40 mL), extracted three times with ethyl acetate (150 mL total). The ethyl acetate fractions we combined and concentrated under reduced pressure to get residue. The residue was purified by flash silica gel chromatography (ISCO®; 1g SepaFlash® Silica Flash Column, eluent of 0~50% PE/EA, Rf = 0.35) to get a O16-2 (1.40 g, 4.01 mmol, 53.8% yield) as

colorless liquid, which was confirmed by HNMR (Figure S10). HNMR: δ 3.80 (br s, 1H), 3.34 - 3.71 (m, 2H), 1.51 - 1.61 (m, 2H), 1.16 - 1.48 (m, 28H), 0.89 (t, J = 6.82 Hz, 3H)

To a mixture of O16-2 (350 mg, 1.00 mmol, 1.00 eq) and Compound 3 (442.5 mg, 1.00 mmol, 1.00 eq) in ACN (2 mL) under dry nitrogen was added KI (182 mg, 1.10 mmol, 1.10 eq) followed by K_2CO_3 (553 mg, 4.01 mmol, 4.00 eq) and the mixture diluted with methoxycyclopentane (900 mg, 9.02 mmol, 1.05 mL, 9.00 eq). The resulting white mixture was heated to 90 ℃ and stirred for 20 hours, TLC (DCM/MeOH = 10/1, PMA) showed the O16-2 (Rf = 0.50) was consumed and one new main spot (Rf = 0.60) was formed. The residue was filtered, the filter was washed by DCM (15.0 mL), the precipitate was filtered off and the filtrate was washed by $NaHCO_3$ (5 mL × 2), dried over Na_2SO_4, the organic phase was concentrated under reduced pressure to get residue. The residue was purified by flash silica gel chromatography (ISCO®;1g SepaFlash® Silica Flash Column, eluent of 0~50% DCM/MeOH, Rf = 0.60) to get HTO16 (150 mg, 209. μmol, 20.8% yield, 99% purity) as yellow oil, which was confirmed by HNMR (Figure S11) and LCMS (Figure S12). HNMR: δ 4.87 (t, J = 6.19 Hz, 1H), 3.60 - 3.74 (m, 3H), 2.72 - 2.87 (m, 1H), 2.56 - 2.67 (m, 2H). 2.47 - 2.55 (m, 2H), 2.37 - 2.45 (m, 1H), 2.23 - 2.31 (m, 2H), 1.57 - 1.67 (m, 2H), 1.21 - 1.53 (m, 65H), 0.88 (t, J = 6.82 Hz, 9H).

Synthesis of mRNA

The coding sequence of Fluc mRNA and ABE mRNA were in sequence information section of Supplemental information. The FLuc and ABE mRNA were produced *in vitro* by T7 RNA polymerase-based transcription from a linearized DNA template containing a 110-nucleotide polyA sequence. Dnase I treatment was applied after transcription. After purification using 2.5 M LiCl solution, mRNA was then heated to 70°C for 5 min and immediately placed on ice for 5 min, after which the mRNA was capped by cap-2'-O-methyltransferase and Vaccinia capping enzyme for 1 h at 37°C. Finally, the processed mRNA was column purified using Monarch® RNA Cleanup Columns (NEB, USA).

Preparation of LNP-mRNA

A microfluidic device was used to prepare the mRNA-LNPs as previously reported[1]. Initially, ionizable lipids, DSPC, cholesterol, and DMG-PEG2000 were dissolved in ethanol in specific molar ratios (50: 10: 38.5: 1.5). The mRNA was dissolved in an acetate buffer solution (6.25 mM). The two solutions were mixed using the microfluidic device at a rate of 12 mL/min (The lipid: mRNA = 1: 3). The resulting mixture was then dialyzed against PBS to remove ethanol. Finally, the mRNA-LNPs were passed through a 0.22 μm filter and then stored at 4°C until further use.

Characterization of LNP-mRNA

The particle size distribution and polydispersity index were analyzed using Malvern Nano ZS90, and the encapsulation efficiency of mRNA was determined using the Quant-iT™ RiboGreen™ RNA Assay[1]. Scientific Inc (USA). The particle size distribution and polydispersity index were analyzed using Malvern Nano ZS90, and the encapsulation efficiency of mRNA was determined using the Quant-iT™ RiboGreen™ RNA Assay. The morphology of LNP was observed using a Cryo-TEM from Thermo Fisher Scientific Inc (USA).

The pKa of each LNP formulation was determined through surface ionization measurements, following a previously reported protocol[2]. In brief, a buffered solution comprising 150 mM sodium chloride, 20 mM sodium phosphate, 20 mM ammonium acetate, and 25 mM ammonium citrate was prepared, and its pH was systematically adjusted within the range of 2 to 12 in increments of 0.5 units. Subsequently, triplicate aliquots of 125 μL from each pH-adjusted solution were combined with 5 μL of each LNP formulation, and the resultant mixtures were dispensed into black 96-well plates. The fluorescent probe 2-(p-toluidinyl) naphthalene-6-sulfonic acid (TNS) was then introduced to each well, achieving a final concentration of 6 μM, followed

by the quantification of fluorescence intensity using a SpectraMax i3x plate reader (Molecular Devices) with an excitation wavelength of 322 nm and an emission wavelength of 431 nm. Employing a linear regression model, the pKa was ascertained as the pH value corresponding to half-maximum fluorescence intensity, indicative of 50% protonation of the LNP formulation.

For mRNA-LNPs stability determination, mRNA-LNPs were stored in PBS buffer (4 ℃) and 10% FBS buffer (37 ℃). The stability was reflected by DLS at different time points. Meanwhile, the *in vivo* delivery efficiency LNPs encapsulating Luc mRNA, which were stored in PBS buffer at 4 ℃ for different days, was determined by biodistribution imaging. The experimental protocol is described in the following section.

The dynamic simulation results of the interactions between HTO12 lipid and RNA molecules were examined using Desmond Software as previous reported, generating MD trajectories spanning 1000 nanoseconds (ns) under conditions of 300 Kelvin (K) and 1 atmosphere (ATM) pressure. Each simulation was encapsulated within a cubic box measuring 10 angstroms (Å) per side and comprised a 10-nucleotide polyA RNA and a positively charged lipid. The system underwent equilibration procedures, with trajectory snapshots captured and analyzed at intervals of 1000 picoseconds (ps)[3].

Expression efficiency and biodistribution of mRNA LNPs *in vivo*

Eight-week-old female C57BL/6J mice were from GemPharmatech Co., Ltd (Nanjing, China) and all mouse investigations were approved by the Institutional Animal Care and Use Committee of Wuhan University. To assess the biodistribution and expression efficiency of those LNPs, Fluc mRNA LNPs were prepared using the same method as described earlier[1]. The Fluc-LNP (0.5 mg/kg) was intravenously injected into the tail vein of the mouse. Then 2 mg of D-Luciferin potassium was injected intraperitoneally after 6 h. Imaging was performed 15 minutes after D-

Luciferin potassium injection to assess luciferase protein expression in live animals or extracted organs (heart, liver, spleen, lung, and kidney) with an exposure time of 1 minute to ensure accurate signal detection. Regions of interest were quantified for further analysis.

Toxicology evaluation

Eight-week-old female C57BL/6J mice, weighing 20 ± 1.5 g, received intravenous administration of empty (blank) SM-102 LNP or HTO12 LNP. The LNP injection dosage was proportional to a mRNA dose of 3 mg/kg or 10 mg/kg. The mice were then sacrificed, and their liver, spleen and blood samples were collected. The concentrations of ALP, ALT, and AST in plasma were measured by the corresponding kits described above. The whole blood components were analyzed using a hematology analyzer (Mindray Biomedical Electronics Co., Ltd., BC-2800VET, Shenzhen, China). H&E staining was performed using liver and spleen tissues.

To assess the potential inflammatory response elicited by HTO12 LNP, mice were intramuscularly administered HTO12 or SM-102 LNP at a dosage of 2 μg mRNA per injection site into the thigh muscles. Twenty-four hours post-injection, muscle tissue samples adjacent to the injection site were collected for RNA extraction. Total RNA was isolated from the tissue, and subsequently, 1 μg of RNA underwent reverse transcription to synthesize cDNA, following the protocol provided by the manufacturer of the respective kits described above. The evaluation of relevant gene expression was conducted *via* RT-qPCR, with *Gapdh* utilized as a reference gene.

Base editing

ABE mRNA was produced by IVT with N1-methyl-pseudouridine modification. sgRNA with phosphorothioate and 2′-O-methyl modification at the 3' and 5' ends was from GenScript (Nanjing, China). The es2-sgRNA sequence was described in sequence information section of Supplemental information. The base editor LNPs were prepared using the previously described

method, with a mass ratio of 2:1 for ABE mRNA to sgRNA, and a mass ratio of 4:1 for HTO12 lipid and total RNA. Eight-week-old female C57BL/6J mice were intravenously administered with blank HTO12 LNP, SM-102 or HTO12 LNPs encapsulating ABE (ABE8.8m & sgRNA LNP) with 3 mg/kg of total RNA dose. Fourteen days after LNP injection, the mice were euthanized, and liver tissues and blood collected. Genomic DNA from the liver was purified using a genomic DNA extraction kit (Magen, Guangzhou, China), following the manufacturer's instructions. The determination of base editing efficiency involved conducting amplicon sequencing analysis, using a minimum confidence cut off of 0.1%[4]. The plasma levels of TC and Pcsk9 were measured using their respective kits.

Statistical analysis

The data were expressed as means ± standard deviation (SD). Statistical analysis was performed using one-way ANOVA. Statistical significance was denoted as *P < 0.05 and **P < 0.01.

Supplemental Sequence Information

ABE8.8m mRNA (Ψ, N1-Methyl-Pseudouridine**)**

AΨGGACΨACAAGGACGACGACGACAAGAΨGGCCCCCAAGAAGAAGAGGAAGGΨGG

GCAΨCCACGGCGΨGCCCGCCGCCAGCGAGGΨGGAGΨΨCAGCCACGAGΨACΨGGAΨ

GAGGCACGCCCΨGACCCΨGGCCAAGAGGGCCAGGGACGAGAGGGAGGΨGCCCGΨG

GGCGCCGΨGCΨGGΨGCΨGAACAACAGGGΨGAΨCGGCGAGGGCΨGGAACAGGGCCA

ΨCGGCCΨGCACGACCCCACCGCCCACGCCGAGAΨCAΨGGCCCΨGAGGCAGGGCGGC

CΨGGΨGAΨGCAGAACΨACAGGCΨGAΨCGACGCCACCCΨGΨACGΨGACCΨΨCGAGC

CCΨGCGΨGAΨGΨGCGCCGGCGCCAΨGAΨCCACAGCAGGAΨCGGCAGGGΨGGΨGΨΨ

CGGCGΨGAGGAACAGCAAGAGGGGCGCCGCCGGCAGCCΨGAΨGAACGΨGCΨGAAC

ΨACCCCGGCAΨGAACCACAGGGΨGGAGAΨCACCGAGGGCAΨCCΨGGCCGACGAGΨ

GCGCCGCCCΨGCΨGΨGCGACΨΨCΨACAGGAΨGCCCAGGCAGGΨGΨΨCAACGCCCA

GAAGAAGGCCCAGAGCAGCAΨCAACAGCGGCGGCAGCAGCGGCGGCAGCAGCGGC
AGCGAGACCCCCGGCACCAGCGAGAGCGCCACCCCCGAGAGCAGCGGCGGCAGCAG
CGGCGGCAGCGACAAGAAGΨACAGCAΨCGGCCΨGGCCAΨCGGCACCAACAGCGΨG
GGCΨGGGCCGΨGAΨCACCGACGAGΨACAAGGΨGCCCAGCAAGAAGΨΨCAAGGΨGC
ΨGGGCAACACCGACAGGCACAGCAΨCAAGAAGAACCΨGAΨCGGCGCCCΨGCΨGΨΨ
CGACAGCGGCGAGACCGCCGAGGCCACCAGGCΨGAAGAGGACCGCCAGGAGGAGG
ΨACACCAGGAGGAAGAACAGGAΨCΨGCΨACCΨGCAGGAGAΨCΨΨCAGCAACGAGA
ΨGGCCAAGGΨGGACGACAGCΨΨCΨΨCCACAGGCΨGGAGGAGAGCΨΨCCΨGGΨGGA
GGAGGACAAGAAGCACGAGAGGCACCCCAΨCΨΨCGGCAACAΨCGΨGGACGAGGΨG
GCCΨACCACGAGAAGΨACCCCACCAΨCΨACCACCΨGAGGAAGAAGCΨGGΨGGACA
GCACCGACAAGGCCGACCΨGAGGCΨGAΨCΨACCΨGGCCCΨGGCCCACAΨGAΨCAA
GΨΨCAGGGGCCACΨΨCCΨGAΨCGAGGGCGACCΨGAACCCCGACAACAGCGACGΨG
GACAAGCΨGΨΨCAΨCCAGCΨGGΨGCAGACCΨACAACCAGCΨGΨΨCGAGGAGAACC
CCAΨCAACGCCAGCGGCGΨGGACGCCAAGGCCAΨCCΨGAGCGCCAGGCΨGAGCAA
GAGCAGGAGGCΨGGAGAACCΨGAΨCGCCCAGCΨGCCCGGCGAGAAGAAGAACGGC
CΨGΨΨCGGCAACCΨGAΨCGCCCΨGAGCCΨGGGCCΨGACCCCCAACΨΨCAAGAGCA
ACΨΨCGACCΨGGCCGAGGACGCCAAGCΨGCAGCΨGAGCAAGGACACCΨACGACGA
CGACCΨGGACAACCΨGCΨGGCCCAGAΨCGGCGACCAGΨACGCCGACCΨGΨΨCCΨG
GCCGCCAAGAACCΨGAGCGACGCCAΨCCΨGCΨGAGCGACAΨCCΨGAGGGΨGAACA
CCGAGAΨCACCAAGGCCCCCCΨGAGCGCCAGCAΨGAΨCAAGAGGΨACGACGAGCA
CCACCAGGACCΨGACCCΨGCΨGAAGGCCCΨGGΨGAGGCAGCAGCΨGCCCGAGAAG
ΨACAAGGAGAΨCΨΨCΨΨCGACCAGAGCAAGAACGGCΨACGCCGGCΨACAΨCGACG
GCGGCGCCAGCCAGGAGGAGΨΨCΨACAAGΨΨCAΨCAAGCCCAΨCCΨGGAGAAGAΨ
GGACGGCACCGAGGAGCΨGCΨGGΨGAAGCΨGAACAGGGAGGACCΨGCΨGAGGAAG
CAGAGGACCΨΨCGACAACGGCAGCAΨCCCCCACCAGAΨCCACCΨGGGCGAGCΨGC
ACGCCAΨCCΨGAGGAGGCAGGAGGACΨΨCΨACCCCΨΨCCΨGAAGGACAACAGGGA

GAAGAΨCGAGAAGAΨCCΨGACCΨΨCAGGAΨCCCCΨACΨACGΨGGGCCCCCΨGGCC
AGGGGCAACAGCAGGΨΨCGCCΨGGAΨGACCAGGAAGAGCGAGGAGACCAΨCACCC
CCΨGGAACΨΨCGAGGAGGΨGGΨGGACAAGGGCGCCAGCGCCCAGAGCΨΨCAΨCGA
GAGGAΨGACCAACΨΨCGACAAGAACCΨGCCCAACGAGAAGGΨGCΨGCCCAAGCAC
AGCCΨGCΨGΨACGAGΨACΨΨCACCGΨGΨACAACGAGCΨGACCAAGGΨGAAGΨACG
ΨGACCGAGGGCAΨGAGGAAGCCCGCCΨΨCCΨGAGCGGCGAGCAGAAGAAGGCCAΨ
CGΨGGACCΨGCΨGΨΨCAAGACCAACAGGAAGGΨGACCGΨGAAGCAGCΨGAAGGAG
GACΨACΨΨCAAGAAGAΨCGAGΨGCΨΨCGACAGCGΨGGAGAΨCAGCGGCGΨGGAGG
ACAGGΨΨCAACGCCAGCCΨGGGCACCΨACCACGACCΨGCΨGAAGAΨCAΨCAAGGA
CAAGGACΨΨCCΨGGACAACGAGGAGAACGAGGACAΨCCΨGGAGGACAΨCGΨGCΨG
ACCCΨGACCCΨGΨΨCGAGGACAGGGAGAΨGAΨCGAGGAGAGGCΨGAAGACCΨACG
CCCACCΨGΨΨCGACGACAAGGΨGAΨGAAGCAGCΨGAAGAGGAGGAGGΨACACCGG
CΨGGGGCAGGCΨGAGCAGGAAGCΨGAΨCAACGGCAΨCAGGGACAAGCAGAGCGGC
AAGACCAΨCCΨGGACΨΨCCΨGAAGAGCGACGGCΨΨCGCCAACAGGAACΨΨCAΨGC
AGCΨGAΨCCACGACGACAGCCΨGACCΨΨCAAGGAGGACAΨCCAGAAGGCCCAGGΨ
GAGCGGCCAGGGCGACAGCCΨGCACGAGCACAΨCGCCAACCΨGGCCGGCAGCCCCG
CCAΨCAAGAAGGGCAΨCCΨGCAGACCGΨGAAGGΨGGΨGGACGAGCΨGGΨGAAGGΨ
GAΨGGGCAGGCACAAGCCCGAGAACAΨCGΨGAΨCGAGAΨGGCCAGGGAGAACCAG
ACCACCCAGAAGGGCCAGAAGAACAGCAGGGAGAGGAΨGAAGAGGAΨCGAGGAGG
GCAΨCAAGGAGCΨGGGCAGCCAGAΨCCΨGAAGGAGCACCCCGΨGGAGAACACCCA
GCΨGCAGAACGAGAAGCΨGΨACCΨGΨACΨACCΨGCAGAACGGCAGGGACAΨGΨAC
GΨGGACCAGGAGCΨGGACAΨCAACAGGCΨGAGCGACΨACGACGΨGGACCACAΨCG
ΨGCCCCAGAGCΨΨCCΨGAAGGACGACAGCAΨCGACAACAAGGΨGCΨGACCAGGAG
CGACAAGAACAGGGGCAAGAGCGACAACGΨGCCCAGCGAGGAGGΨGGΨGAAGAAG
AΨGAAGAACΨACΨGGAGGCAGCΨGCΨGAACGCCAAGCΨGAΨCACCCAGAGGAAGΨ
ΨCGACAACCΨGACCAAGGCCGAGAGGGGCGGCCΨGAGCGAGCΨGGACAAGGCCGG

CΨΨCAΨCAAGAGGCAGCΨGGΨGGAGACCAGGCAGAΨCACCAAGCACGΨGGCCCAG

AΨCCΨGGACAGCAGGAΨGAACACCAAGΨACGACGAGAACGACAAGCΨGAΨCAGGG

AGGΨGAAGGΨGAΨCACCCΨGAAGAGCAAGCΨGGΨGAGCGACΨΨCAGGAAGGACΨ

ΨCCAGΨΨCΨACAAGGΨGAGGGAGAΨCAACAACΨACCACCACGCCCACGACGCCΨA

CCΨGAACGCCGΨGGΨGGGCACCGCCCΨGAΨCAAGAAGΨACCCCAAGCΨGGAGAGC

GAGΨΨCGΨGΨACGGCGACΨACAAGGΨGΨACGACGΨGAGGAAGAΨGAΨCGCCAAG

AGCGAGCAGGAGAΨCGGCAAGGCCACCGCCAAGΨACΨΨCΨΨCΨACAGCAACAΨCA

ΨGAACΨΨCΨΨCAAGACCGAGAΨCACCCΨGGCCAACGGCGAGAΨCAGGAAGAGGCC

CCΨGAΨCGAGACCAACGGCGAGACCGGCGAGAΨCGΨGΨGGGACAAGGGCAGGGAC

ΨΨCGCCACCGΨGAGGAAGGΨGCΨGAGCAΨGCCCCAGGΨGAACAΨCGΨGAAGAAGA

CCGAGGΨGCAGACCGGCGGCΨΨCAGCAAGGAGAGCAΨCCΨGCCCAAGAGGAACAG

CGACAAGCΨGAΨCGCCAGGAAGAAGGACΨGGGACCCCAAGAAGΨACGGCGGCΨΨC

GACAGCCCCACCGΨGGCCΨACAGCGΨGCΨGGΨGGΨGGCCAAGGΨGGAGAAGGGCA

AGAGCAAGAAGCΨGAAGAGCGΨGAAGGAGCΨGCΨGGGCAΨCACCAΨCAΨGGAGA

GGAGCAGCΨΨCGAGAAGAACCCCAΨCGACΨΨCCΨGGAGGCCAAGGGCΨACAAGGA

GGΨGAAGAAGGACCΨGAΨCAΨCAAGCΨGCCCAAGΨACAGCCΨGΨΨCGAGCΨGGAG

AACGGCAGGAAGAGGAΨGCΨGGCCAGCGCCGGCGAGCΨGCAGAAGGGCAACGAGC

ΨGGCCCΨGCCCAGCAAGΨACGΨGAACΨΨCCΨGΨACCΨGGCCAGCCACΨACGAGAA

GCΨGAAGGGCAGCCCCGAGGACAACGAGCAGAAGCAGCΨGΨΨCGΨGGAGCAGCAC

AAGCACΨACCΨGGACGAGAΨCAΨCGAGCAGAΨCAGCGAGΨΨCAGCAAGAGGGΨGA

ΨCCΨGGCCGACGCCAACCΨGGACAAGGΨGCΨGAGCGCCΨACAACAAGCACAGGGA

CAAGCCCAΨCAGGGAGCAGGCCGAGAACAΨCAΨCCACCΨGΨΨCACCCΨGACCAAC

CΨGGGCGCCCCCGCCGCCΨΨCAAGΨACΨΨCGACACCACCAΨCGACAGGAAGAGGΨ

ACACCAGCACCAAGGAGGΨGCΨGGACGCCACCCΨGAΨCCACCAGAGCAΨCACCGG

CCΨGΨACGAGACCAGGAΨCGACCΨGAGCCAGCΨGGGCGGCGACAAGCGGCCCGCC

GCCACCAAGAAGGCCGGCCAGGCCAAGAAGAAGAAGΨGA

Fluc mRNA

AΨGGAAGACGCCAAAAACAΨAAAGAAAGGCCCGGCGCCAΨΨCΨAΨCCGCΨGGAAG
AΨGGAACCGCΨGGAGAGCAACΨGCAΨAAGGCΨAΨGAAGAGAΨACGCCCΨGGΨΨCC
ΨGGAACAAΨΨGCΨΨΨΨACAGAΨGCACAΨAΨCGAGGΨGGACAΨCACΨΨACGCΨGA
GΨACΨΨCGAAAΨGΨCCGΨΨCGGΨΨGGCAGAAGCΨAΨGAAACGAΨAΨGGGCΨGAA
ΨACAAAΨCACAGAAΨCGΨCGΨAΨGCAGΨGAAAACΨCΨCΨΨCAAΨΨCΨΨΨAΨGCCG
GΨGΨΨGGGCGCGΨΨAΨΨΨAΨCGGAGΨΨGCAGΨΨGCGCCCGCGAACGACAΨΨΨAΨ
AAΨGAACGΨGAAΨΨGCΨCAACAGΨAΨGGGCAΨΨΨCGCAGCCΨACCGΨGGΨGΨΨC
GΨΨΨCCAAAAAGGGGΨΨGCAAAAAAΨΨΨΨGAACGΨGCAAAAAAAGCΨCCCAAΨC
AΨCCAAAAAAΨΨAΨΨAΨCAΨGGAΨΨCΨAAAACGGAΨΨACCAGGGAΨΨΨCAGΨCG
AΨGΨACACGΨΨCGΨCACAΨCΨCAΨCΨACCΨCCCGGΨΨΨΨAAΨGAAΨACGAΨΨΨΨG
ΨGCCAGAGΨCCΨΨCGAΨAGGGACAAGACAAΨΨGCACΨGAΨCAΨGAACΨCCΨCΨGG
AΨCΨACΨGGΨCΨGCCΨAAAGGΨGΨCGCΨCΨGCCΨCAΨAGAACΨGCCΨGCGΨGAGA
ΨΨCΨCGCAΨGCCAGAGAΨCCΨAΨΨΨΨΨGGCAAΨCAAAΨCAΨΨCCGGAΨACΨGCGA
ΨΨΨΨAAGΨGΨΨGΨΨCCAΨΨCCAΨCACGGΨΨΨΨGGAAΨGΨΨΨACΨACACΨCGGAΨ
AΨΨΨGAΨAΨGΨGGAΨΨΨCGAGΨCGΨCΨΨAAΨGΨAΨAGAΨΨΨGAAGAAGAGCΨGΨ
ΨΨCΨGAGGAGCCΨΨCAGGAΨΨACAAGAΨΨCAAAGΨGCGCΨGCΨGGΨGCCAACCCΨ
AΨΨCΨCCΨΨCΨΨCGCCAAAAGCACΨCΨGAΨΨGACAAAΨACGAΨΨΨAΨCΨAAΨΨΨ
ACACGAAAΨΨGCΨΨCΨGGΨGGCGCΨCCCCΨCΨCΨAAGGAAGΨCGGGGAAGCGGΨΨ
GCCAAGAGGΨΨCCAΨCΨGCCAGGΨAΨCAGGCAAGGAΨAΨGGGCΨCACΨGAGACΨA
CAΨCAGCΨAΨΨCΨGAΨΨACACCCGAGGGGGAΨGAΨAAACCGGGCGCGGΨCGGΨAA
AGΨΨGΨΨCCAΨΨΨΨΨΨGAAGCGAAGGΨΨGΨGGAΨCΨGGAΨACCGGGAAAACGCΨ
GGGCGΨΨAAΨCAAAGAGGCGAACΨGΨGΨGΨGAGAGGΨCCΨAΨGAΨΨAΨGΨCCGG
ΨΨAΨGΨAAACAAΨCCGGAAGCGACCAACGCCΨΨGAΨΨGACAAGGAΨGGAΨGGCΨ
ACAΨΨCΨGGAGACAΨAGCΨΨACΨGGGACGAAGACGAACACΨΨCΨΨCAΨCGΨΨGAC

CGCCΨGAAGΨCΨCΨGAΨΨAAGΨACAAAGGCΨAΨCAGGΨGGCΨCCCGCΨGAAΨΨGG
AAΨCCAΨCΨΨGCΨCCAACACCCCAACAΨCΨΨCGACGCAGGΨGΨCGCAGGΨCΨΨCC
CGACGAΨGACGCCGGΨGAACΨΨCCCGCCGCCGΨΨGΨΨGΨΨΨGGAGCACGGAAAG
ACGAΨGACGGAAAAAGAGAΨCGΨGGAΨΨACGΨCGCCAGΨCAAGΨAACAACCGCGA
AAAAGΨΨGCGCGGAGGAGΨΨGΨGΨΨΨGΨGGACGAAGΨACCGAAAGGΨCΨΨACCG
GAAAACΨCGACGCAAGAAAAAΨCAGAGAGAΨCCΨCAΨAAAGGCCAAGAAGGGCGG
AAAGAΨCGCCGΨGΨAA

es2 sgRNA sequence (chemical modifications: m, 2’-OMe. *, phosphorothioate)

mC*mC*mC*AUACCUUGGAGCAACGGGUUUUAGAGCUAGAAAUAGCAAGUUAAAA
UAAGGCUAGUCCGUUAUCAACUUGAAAAAGUGGCACCGAGUCGGUGCUU*mU*mU
*mU

Supplemental Figure

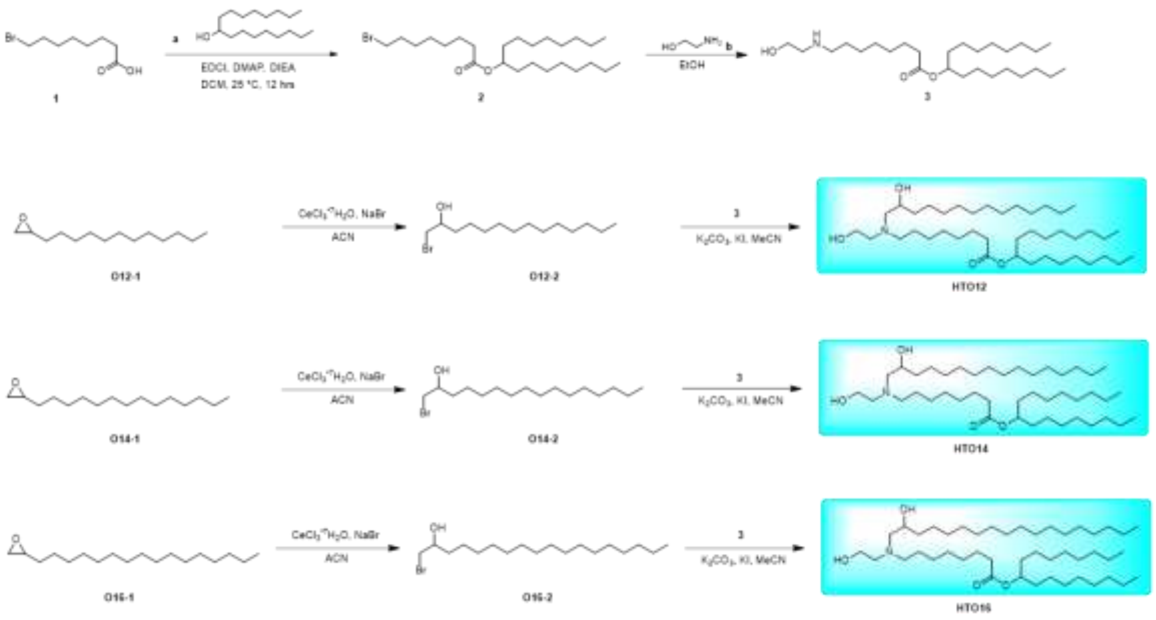

Supplementary Figure 1 Synthesis route of HTO12, HTO14 and HTO16.

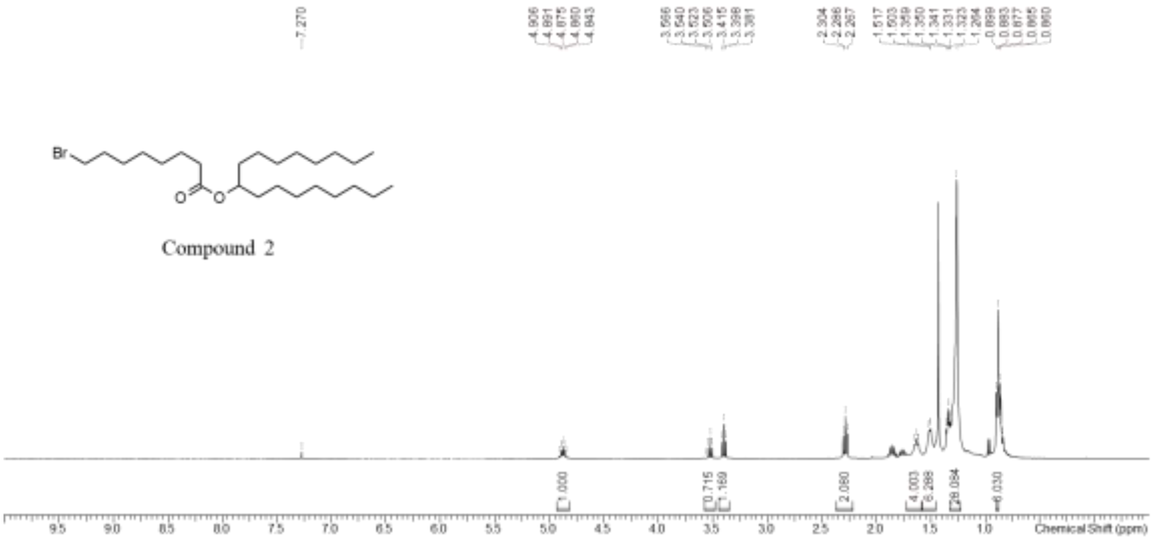

Supplementary Figure 2 Identification of compound 2 using ^{1}H NMR.

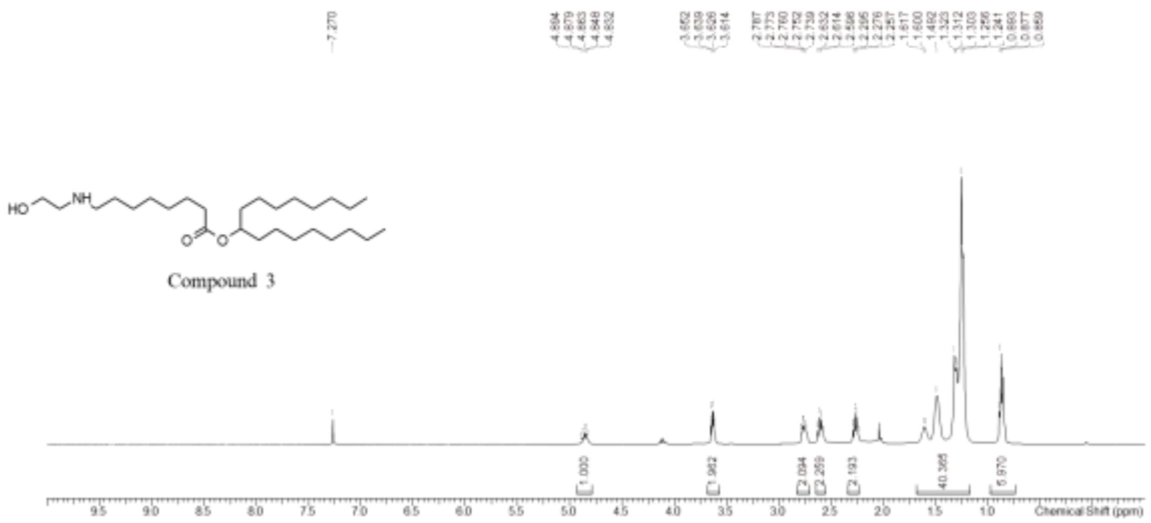

Supplementary Figure 3 Identification of compound 3 using ^{1}H NMR.

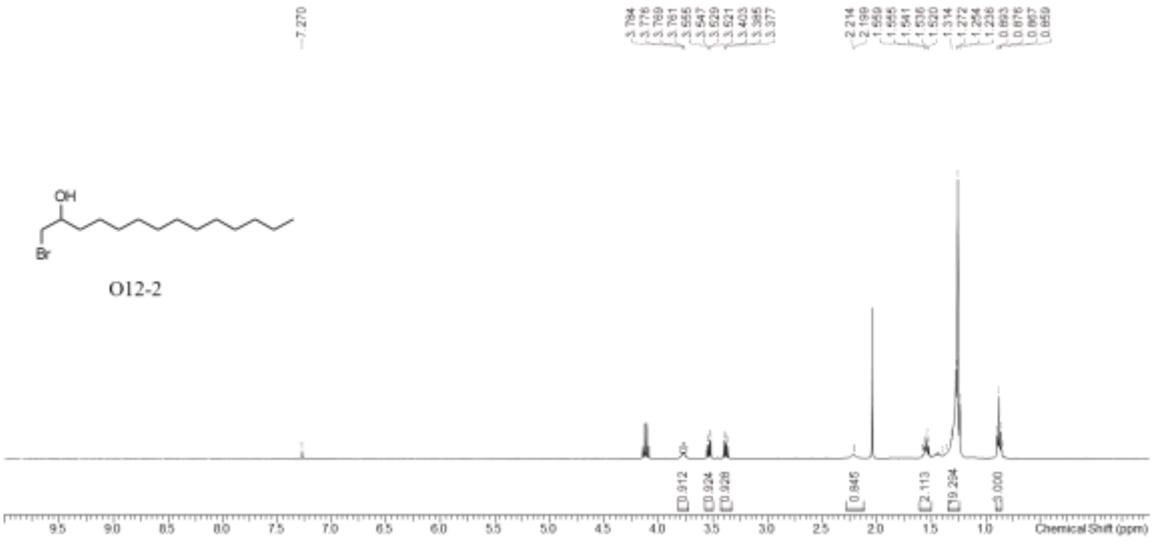

Supplementary Figure 4 Identification of compound O12-2 using ^{1}H NMR.

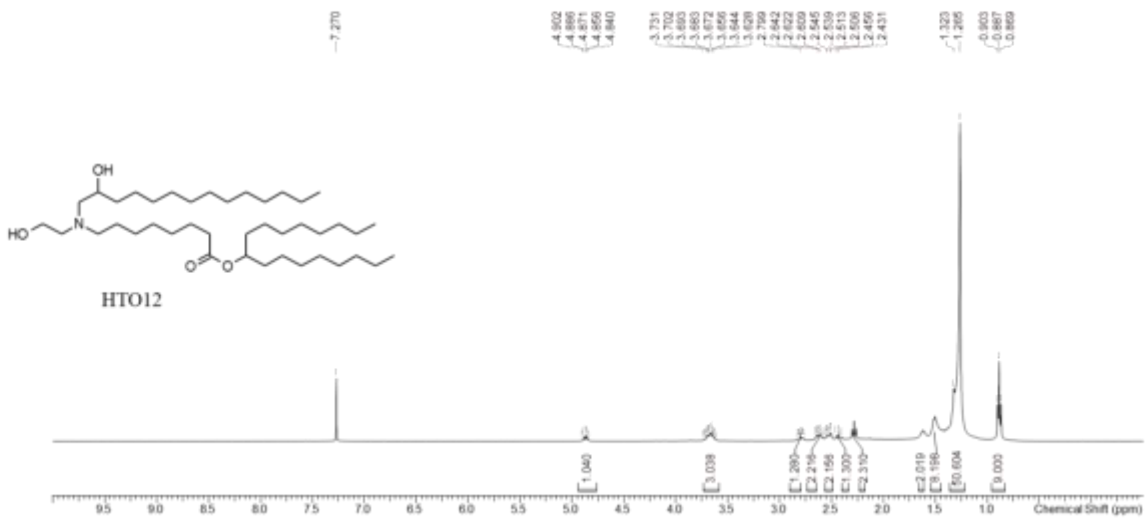

Supplementary Figure 5 Identification of HTO12 using 1H NMR.

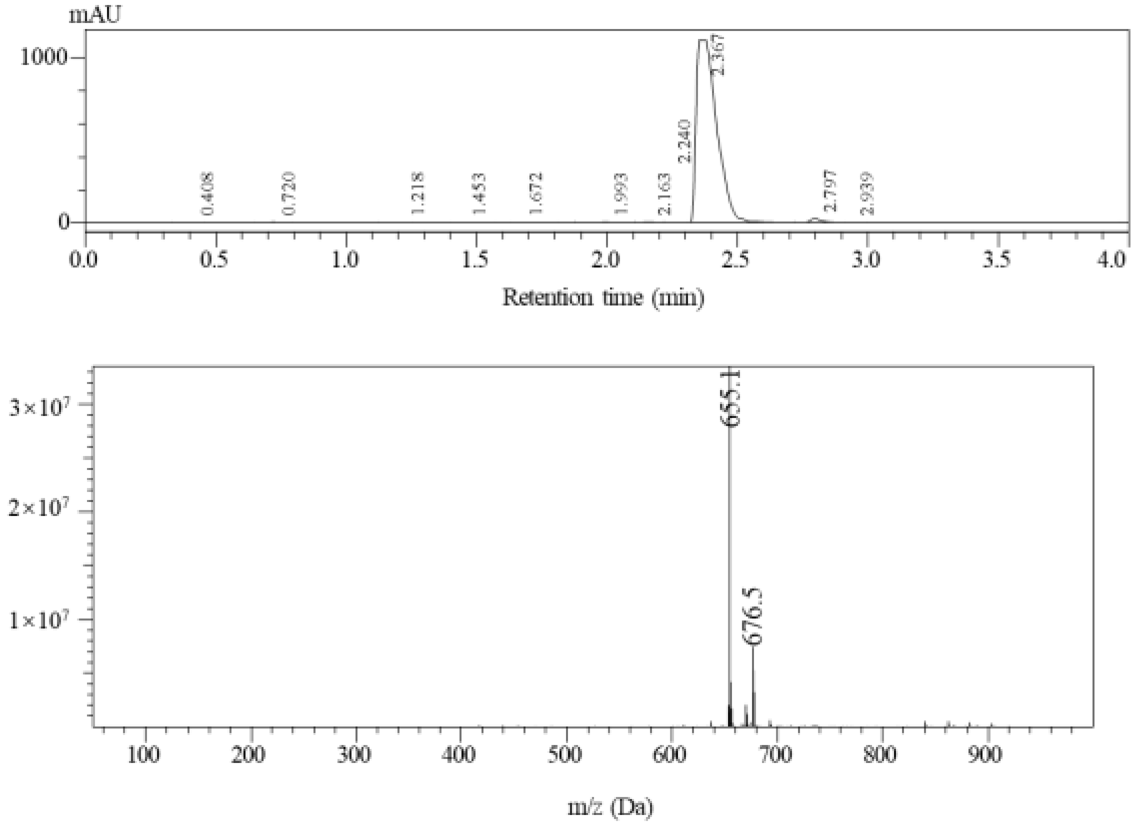

MS (ESI) for HTO12

Calculated: 653.63

Found: 655.1

Supplementary Figure 6 LC-MS spectra of HTO12

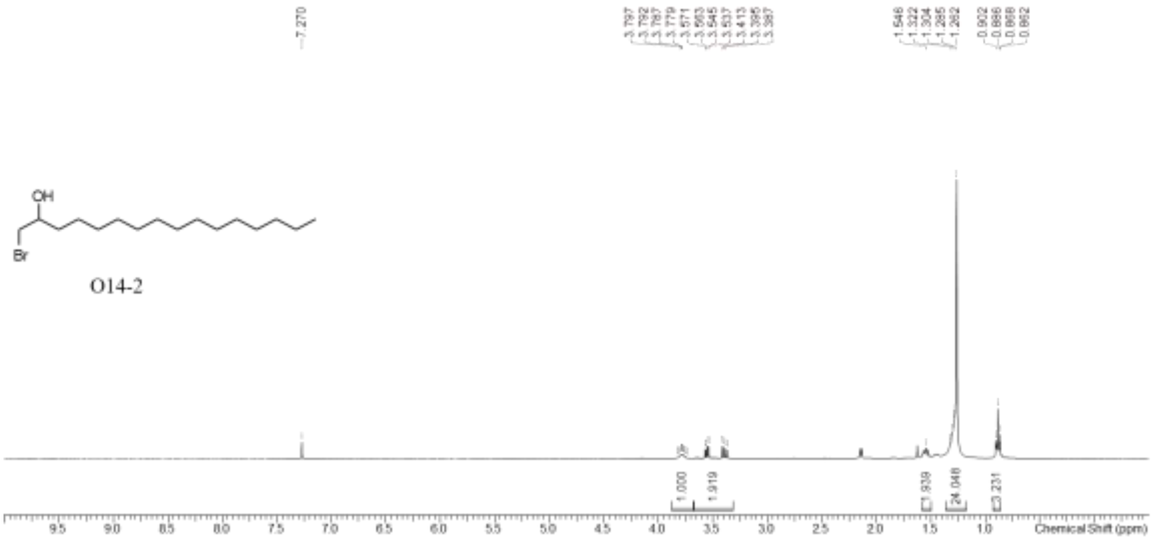

Supplementary Figure 7 Identification of compound O14-2 using ^{1}H NMR.

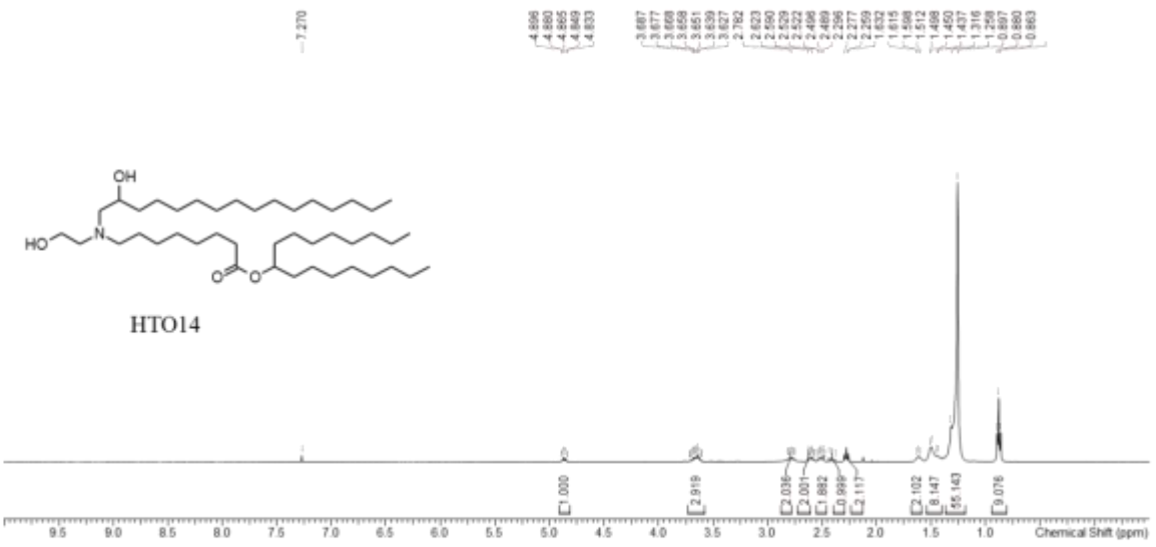

Supplementary Figure 8 Identification of HTO14 using ^{1}H NMR.

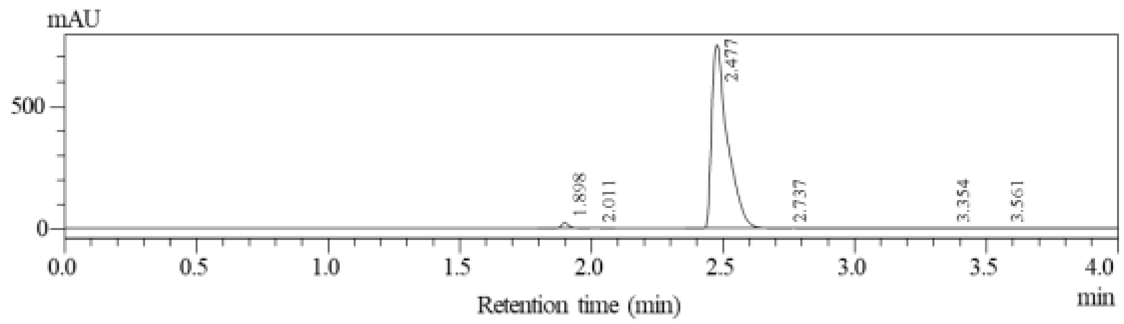

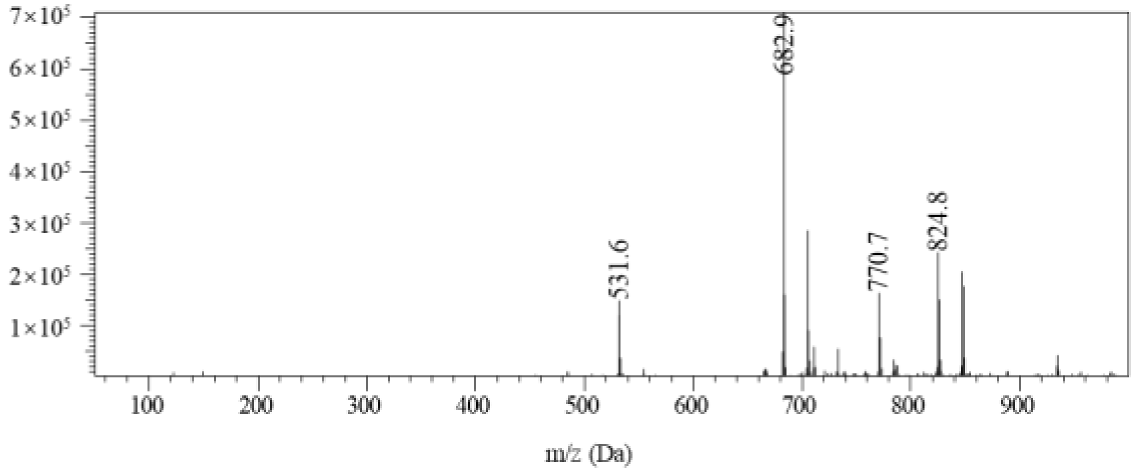

MS (ESI) for HTO14

Calculated: 681.66

Found: 682.9

Supplementary Figure 9 LC-MS spectra of HTO14.

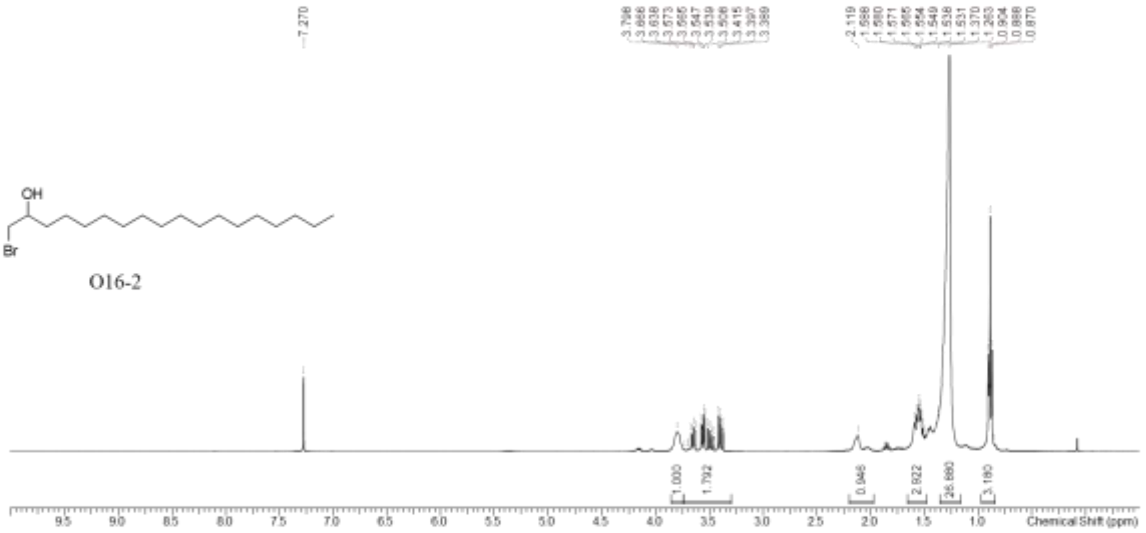

Supplementary Figure 10 Identification of compound O16-2 using ^{1}H NMR.

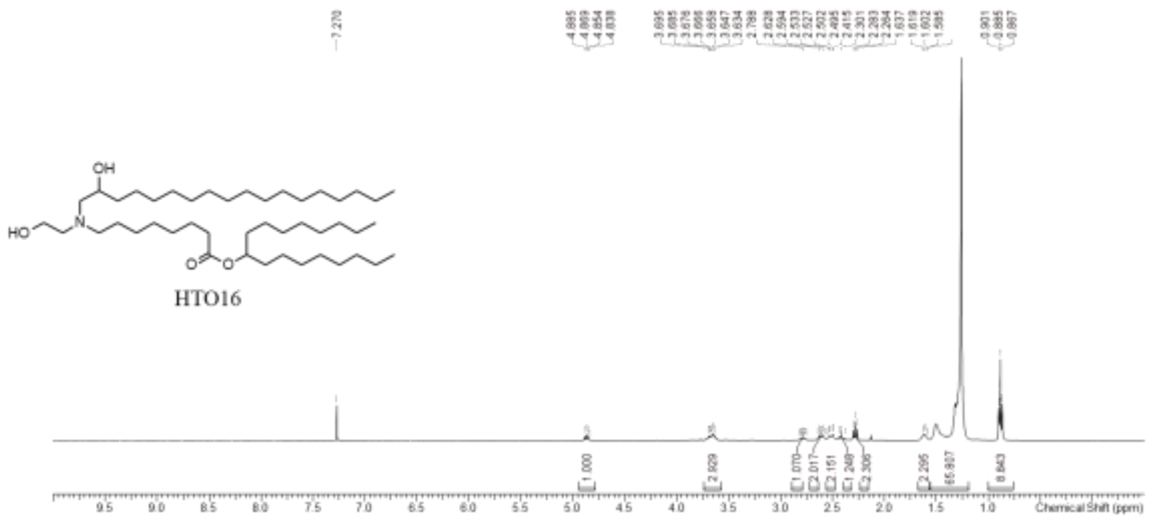

Supplementary Figure 11 Identification of HTO16 using ^{1}H NMR.

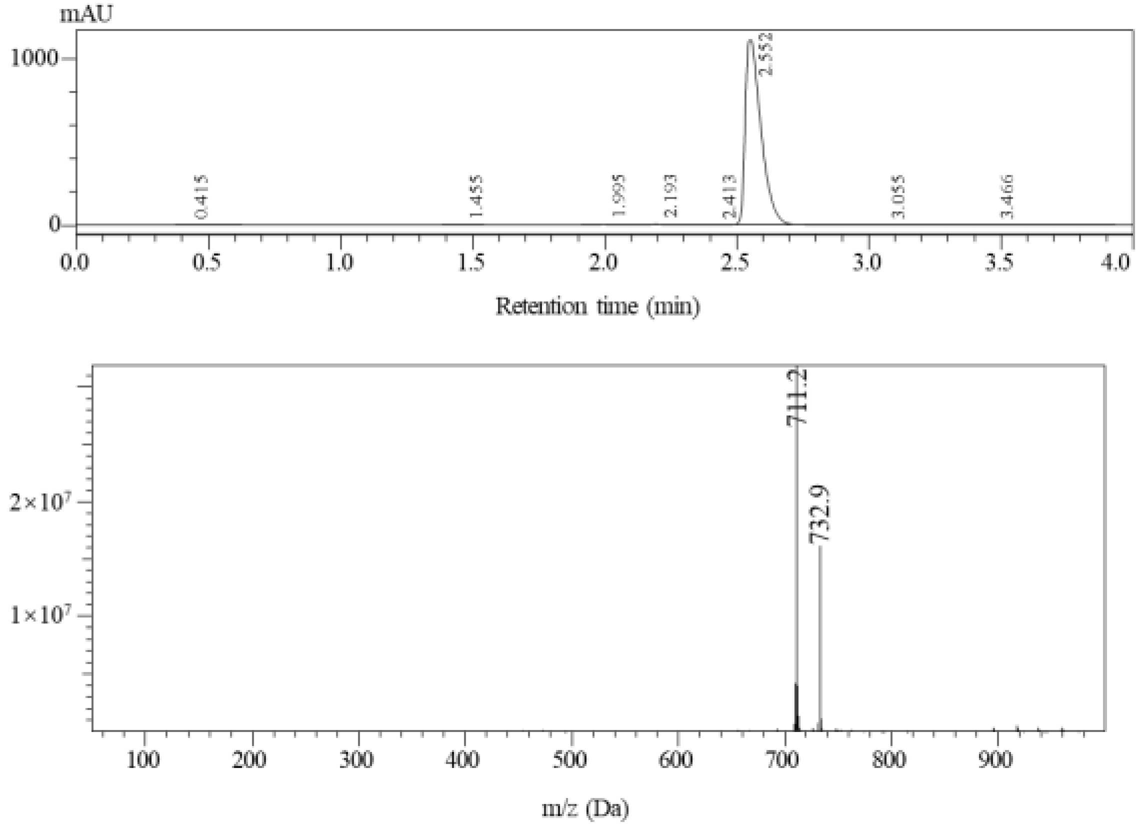

MS (ESI) for HTO16

Calculated: 709.69

Found: 711.2

Supplementary Figure 12 LC-MS spectra of HTO16.

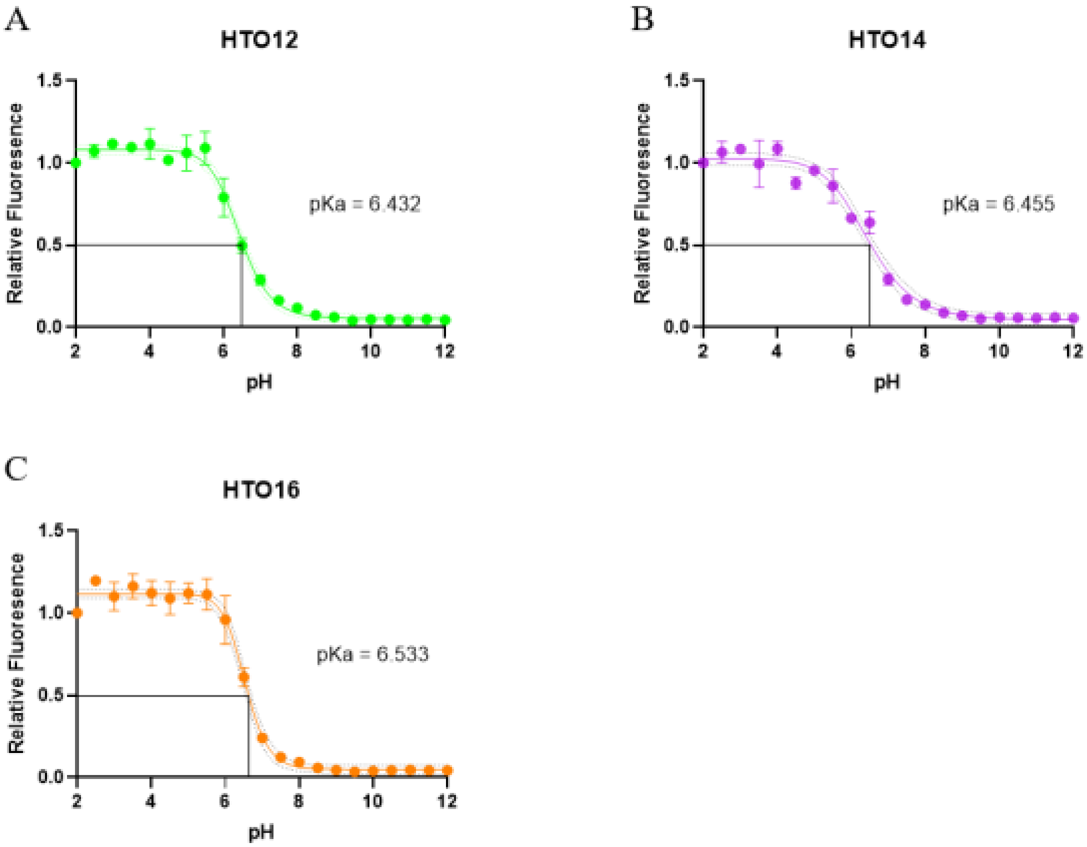

Supplementary Figure 13 The determination of pKa for HTO12 (A), HTO14 (B) and HTO16 (C) using TNS probe.

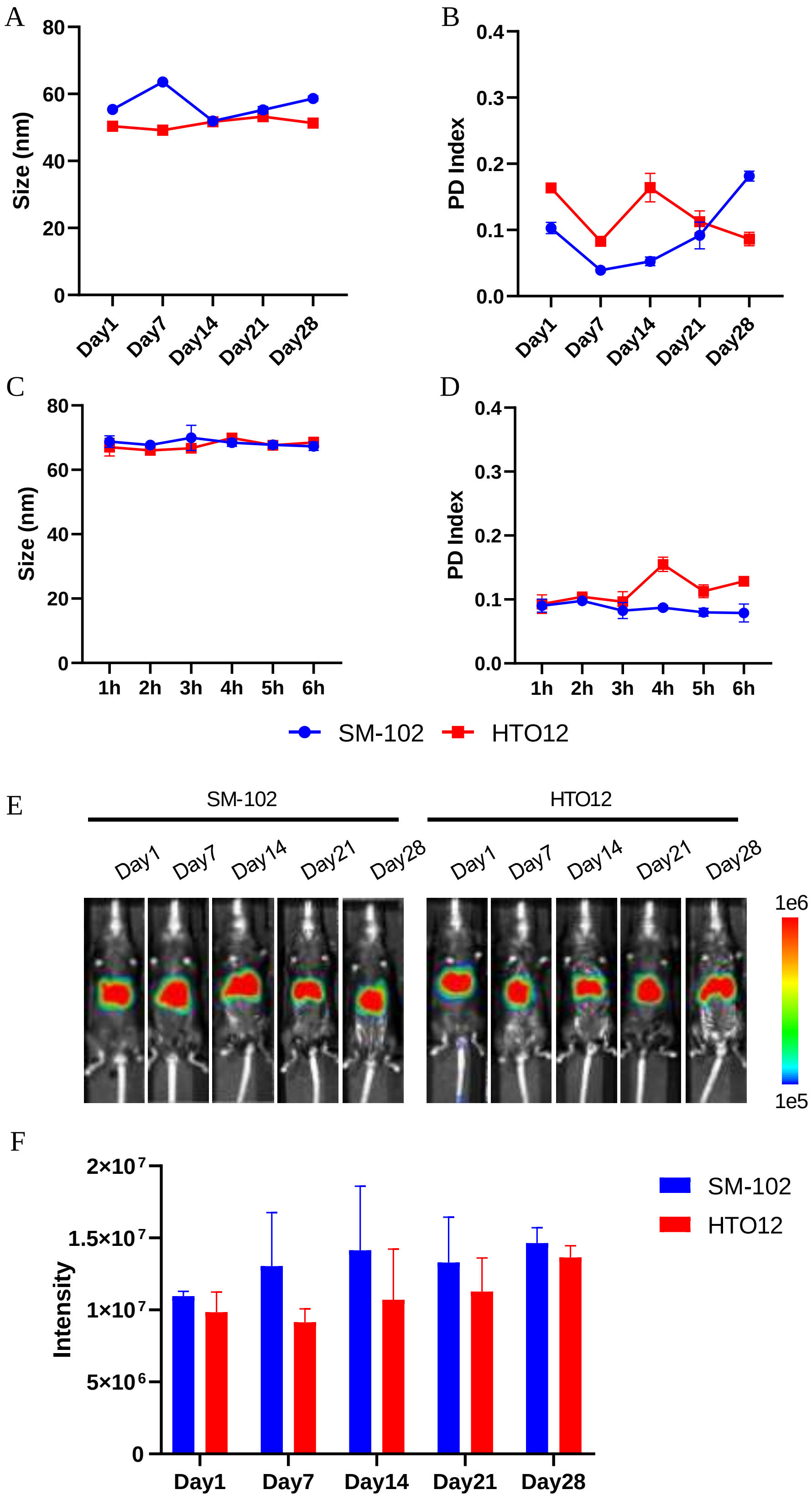
A
Size (nm)
Day1
Day7
Day14
Day21
Day28
B
PD Index
C
Size (nm)
1h
2h
3h
4h
5h
6h
D
PD Index
SM-102
HTO12
E
SM-102
HTO12
1e6
1e5
F
Intensity
2×10^7
1.5×10^7
1×10^7
5×10^6
0
Day1
Day7
Day14
Day21
Day28
SM-102
HTO12

Supplementary Figure 14 Chemical stability of SM-102 and HTO12 LNP in (A-B) PBS at 4°C and (C-D) FBS at 37°C. (E) *In vivo* whole-body luminescence imaging and (F) relative quantified average luminescence of of SM-102 and HTO12 LNPs stored at 4°C for a certain period of time at a dose of 0.5mg/kg. Each point represents the mean ± SD (n = 3).

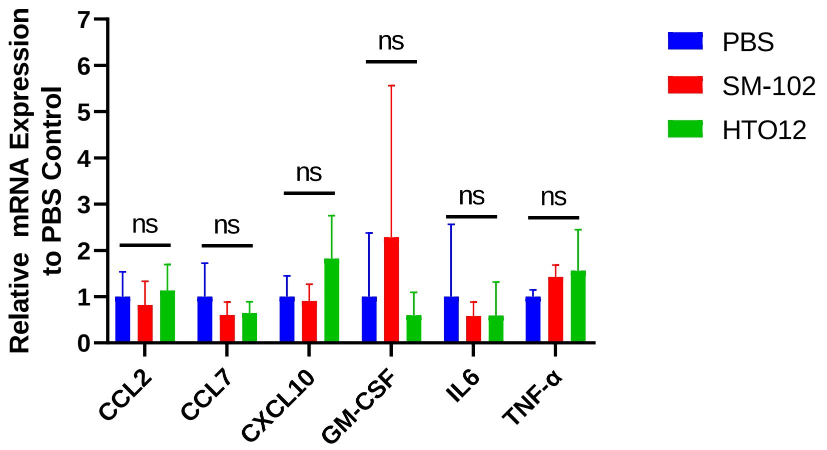

Supplementary Figure 15 Relative mRNA expression of chemokines and inflammatory factors after 24 h of intramuscular administration of SM-102 LNP and HTO12 LNP at a dosage of 0.1 mg/kg into the thigh muscles. Each point represents the mean ± SD (n = 3).

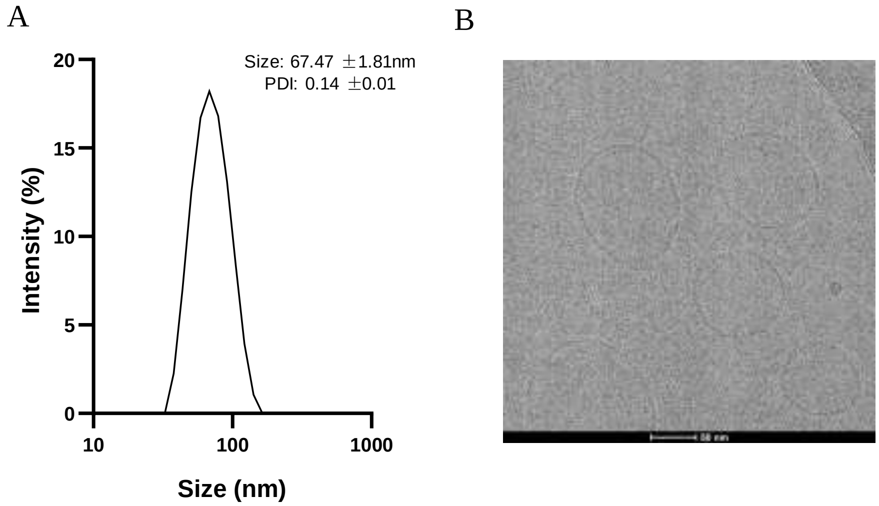

Supplementary Figure 16 Characterization of ABE mRNA/sgRNA HTO12 LNPs including (A) size and (B) typic cyto-TEM image. Scare bar: 50 µm.

Supplemental Table

Supplemental Table S1 Characterization of size *by intensity*, PDI and zeta potential for SM-102, HTO12, HTO14 and HTO16 with optimal ionizable lipid: mRNA weight ratio.

	Optimal ionizable lipid mRNA weight ratio	Size (nm)	PDI	Zeta-potential (mV)
SM-102	10:1	65.51 ± 0.89	0.08 ± 0.01	-10.95 ± 0.84
HTO12	4:1	62.84 ± 0.62	0.10 ± 0.01	-9.10 ± 1.06
HTO14	12:1	61.09 ± 0.32	0.11 ± 0.002	-9.49 ± 0.74
HTO16	10:1	64.42 ± 0.19	0.10 ± 0.01	-9.98 ± 0.87

Supplemental Table S2 NGS primers

Prime name	Sequence (5' to 3')
NGS-ES2-F	ACACTCTTTCCCTACACGACGCTCTTCCGATCTNNNNNgcagctgagcacta agtctt
NGS-ES2-R	GTGACTGGAGTTCAGACGTGTGCTCTTCCGATCTNNNNNaggacgaggat ggagattatga

Supplemental Table S3 qPCR primers

Name	Forward (5' to 3')	Reverse (5' to 3')
Ccl2	TTAAAAACCTGGATCGGAACCAA	GCATTAGCTTCAGATTTACGGGT
Ccl7	GCTGCTTTCAGCATCCAAGTG	CCAGGGACACCGACTACTG
Cxcl10	CCAAGTGCTGCCGTCATTTTC	GGCTCGCAGGGATGATTTCAA
Gm-Csf	GGCCTTGGAAGCATGTAGAGG	GGAGAACTCGTTAGAGACGACTT
Il-6	TAGTCCTTCCTACCCCAATTTCC	TTGGTCCTTAGCCACTCCTTC
Tnf-α	CCCTCACACTCAGATCATCTTCT	GCTACGACGTGGGCTACAG
Gapdh	AGGTCGGTGTGAACGGATTTG	TGTAGACCATGTAGTTGAGGTCA

References

1 Yi JZ, Lei XL, Guo FT, *et al.*, Co-delivery of Cas9 mRNA and guide RNAs edits hepatitis B virus episomal and integration DNA in mouse and tree shrew models, *Antivir Res* 2023; **215**.

2 Hu B, Li B, Li K, *et al.*, Thermostable ionizable lipid-like nanoparticle (iLAND) for RNAi treatment of hyperlipidemia, *Sci Adv* 2022; **8**.

3 Cornebise M, Narayanan E, Xia Y, *et al.*, Discovery of a Novel Amino Lipid That Improves Lipid Nanoparticle Performance through Specific Interactions with mRNA, *Adv Funct Mater* 2022; **32**.

4 Musunuru K, Chadwick AC, Mizoguchi T, *et al.*, In vivo CRISPR base editing of PCSK9 durably lowers cholesterol in primates, *Nature* 2021; **593:** 429.
